# Comparing optimized exoskeleton assistance of the hip, knee, and ankle in single and multi-joint configurations

**DOI:** 10.1017/wtc.2021.14

**Published:** 2021-11-24

**Authors:** Patrick W. Franks, Gwendolyn M. Bryan, Russell M. Martin, Ricardo Reyes, Ava C. Lakmazaheri, Steven H. Collins

**Affiliations:** Department of Mechanical Engineering, Stanford University, Stanford, California, USA

**Keywords:** biomechanics, exoskeleton, human-in-the-loop optimization

## Abstract

Exoskeletons that assist the hip, knee, and ankle joints have begun to improve human mobility, particularly by reducing the metabolic cost of walking. However, direct comparisons of optimal assistance of these joints, or their combinations, have not yet been possible. Assisting multiple joints may be more beneficial than the sum of individual effects, because muscles often span multiple joints, or less effective, because single-joint assistance can indirectly aid other joints. In this study, we used a hip–knee–ankle exoskeleton emulator paired with human-in-the-loop optimization to find single-joint, two-joint, and whole-leg assistance that maximally reduced the metabolic cost of walking. Hip-only and ankle-only assistance reduced the metabolic cost of walking by 26 and 30% relative to walking in the device unassisted, confirming that both joints are good targets for assistance (*N* = 3). Knee-only assistance reduced the metabolic cost of walking by 13%, demonstrating that effective knee assistance is possible (*N* = 3). Two-joint assistance reduced the metabolic cost of walking by between 33 and 42%, with the largest improvements coming from hip-ankle assistance (*N* = 3). Assisting all three joints reduced the metabolic cost of walking by 50%, showing that at least half of the metabolic energy expended during walking can be saved through exoskeleton assistance (*N* = 4). Changes in kinematics and muscle activity indicate that single-joint assistance indirectly assisted muscles at other joints, such that the improvement from whole-leg assistance was smaller than the sum of its single-joint parts. Exoskeletons can assist the entire limb for maximum effect, but a single well-chosen joint can be more efficient when considering additional factors such as weight and cost.

## Impact Statement

Exoskeletons could make walking easier for people, from military personnel to older adults. They can reduce the energetic cost of walking, but we still do not know the best way to assist walking. Which leg joints should exoskeletons assist? What torques should they apply? What is the greatest improvement we could expect? To study this, we optimized hip–knee–ankle exoskeleton assistance for each joint individually, for two-joint combinations, and for the whole leg. We found that assisting the whole leg reduced the energy cost of walking by 50%, double the state-of-the-art. We also found that while assisting the whole-leg was most effective, assisting a single joint may be more efficient when considering device mass. These findings will help exoskeleton designers choose which joints to assist.

## Introduction

Lower limb exoskeletons can assist human locomotion by reducing the metabolic cost of walking. These devices have the potential to restore ambulatory ability lost from age or disability, and increase maximum performance for high-activity users like first responders, military personnel, or athletes. One important way exoskeletons can help is by reducing the metabolic cost of walking, which is the amount of biochemical energy consumed to produce walking at a given speed (Das Gupta et al., 2019). Humans tend to move in ways that minimize metabolic cost (Zarrugh et al., 1974; Bertram and Ruina, 2001; Donelan et al., 2001; Sanchez et al., 2020), indicating its importance. Reducing the metabolic cost of walking is considered the gold standard for evaluating performance-augmenting exoskeletons (Young and Ferris, 2017; Sawicki et al., 2020). By reducing metabolic cost, exoskeletons could help achieve related mobility outcomes like increasing the user’s walking speed or decreasing fatigue.

Exoskeletons have improved walking by reducing metabolic cost, but larger improvements may be necessary for widespread adoption of exoskeleton products. Reductions in metabolic cost have been demonstrated from assisting one or two joints with both tethered and mobile exoskeletons (Malcolm et al., 2013; Mooney et al., 2014; Collins et al., 2015; Seo et al., 2016; Quinlivan et al., 2017; Zhang et al., 2017; Ding et al., 2018; Lee et al., 2018; Malcolm et al., 2018; Lim et al., 2019; MacLean and Ferris, 2019; Cao et al., 2020; Sawicki et al., 2020). The largest metabolic cost reductions have been around 18% relative to walking in no exoskeleton using hip-only assistance on a mobile device (Ding et al., 2018; Lim et al., 2019) and 24% relative to walking in an exoskeleton with no torques applied using tethered bilateral ankle assistance (Zhang et al., 2017). Despite demonstrated improvements, exoskeletons are in a nascent stage of commercial development, and widespread adoption has not yet occurred (Young and Ferris, 2017). To promote adoption, devices may need larger improvements that offset the negative impacts of exoskeletons, such as worn mass, bulkiness, or cost. The added mass of wearing a device, which can vary with device design, imposes a metabolic penalty, which could eliminate small benefits. Users may not be able to sense if the exoskeleton is assisting them because the largest improvements in the field are similar to the just-noticeable difference for metabolic cost (around 20%, Medrano et al., 2020). Improving our scientific and engineering understanding of exoskeleton assistance could deliver larger benefits that may lead to widespread adoption of these devices.

Whole-leg exoskeleton assistance could produce the largest improvements to walking performance. Assisting all of the lower-limb joints simultaneously seems likely to yield the largest energy savings because the hips, knees and ankles all significantly contribute to biological energy consumption during walking (Winter, 1991; Farris and Sawicki, 2012). Simulations of exoskeleton assistance also indicate that whole-leg devices should be most effective (Uchida et al., 2016; Dembia et al., 2017; Franks et al., 2020). Unfortunately, there has been limited testing of whole-leg assistance for able-bodied users (Zoss et al., 2006), and these devices have not yet reduced metabolic cost (Gregorczyk et al., 2010). With improvements to exoskeleton hardware and control, larger benefits might be realizable.

While whole-body assistance may produce larger benefits, single-joint assistance may be more efficient. Assisting just one joint could lead to a smaller, lighter and more cost-effective device, which could result in better net improvements. While a variety of single-joint devices have been tested, there has not been a well-controlled comparison between them owing to differences in actuator capabilities and control. Each device has had different limits on torque and power (Bryan et al., 2020), many below previously identified optimal values (Zhang et al., 2017), and larger torque and power capacity are associated with larger reductions in metabolic cost (Quinlivan et al., 2017; Ding et al., 2018). Most devices have not used control that has been systematically optimized for the participant, which can improve performance by as much as a factor of five (Jackson and Collins, 2015; Zhang et al., 2017) and has led to the largest improvements in the metabolic cost of walking (Zhang et al., 2017; Ding et al., 2018). A direct comparison of optimized single-joint assistance in a high-torque, high-power exoskeleton would be useful to designers as they choose which joint, and how many joints, to assist.

A comparison of single-joint and multi-joint assistance would also provide scientific insights into the biomechanics of walking. A large portion of human leg musculature is biarticular (van Ingen Schenau et al., 1987), comprising muscles that span two joints. Biarticular muscles might be more effectively assisted by multi-joint exoskeletons, leading to a total benefit beyond the sum of assisting each joint individually. Some experiments have suggested that multi-joint assistance might be more effective than single-joint assistance (Ding et al., 2017; Malcolm et al., 2018). Alternatively, adding assistance at other joints may have diminishing returns. Users might adapt their walking pattern to maximize the benefit from assistance at a single-joint, thereby indirectly benefiting muscles at other joints, which may make it relatively less effective to assist additional joints. Some experiments have shown indications of such indirect assistance (Lenzi et al., 2013; Mooney and Herr, 2016; Jackson et al., 2017). A well-controlled comparison allowing observations of how users respond to different types of assistance would help us to develop improved models of biomechanical and neural adaptation to exoskeletons.

The purpose of this study was to find the single-joint, two-joint, and whole-leg exoskeleton torques that minimized the metabolic cost of walking and to understand how effective each device architecture is at assisting walking. We used a tethered hip–knee–ankle exoskeleton emulator that can assist hip flexion and extension, knee flexion and extension, and ankle plantarflexion of both legs (Bryan et al., 2020). This lab-bound device uses off-board motors and power to apply large torques to the user while minimizing worn mass. This allows for comparison of different actuation schemes without the difficulty of designing product-like mobile devices, which enables us to identify optimal actuation parameters to inform design specifications of future untethered exoskeletons. We paired this tool with human-in-the-loop optimization, a process where the control of the exoskeleton is updated in real time based on biomechanical measurements of the user (Felt et al., 2015; Koller et al., 2016; Kim et al., 2017; Zhang et al., 2017). We performed experiments optimizing and comparing assistance at the hips, knees, and ankles, individually, in pairs, and with all three joints assisted simultaneously. We optimized each assistance pattern to reduce the measured metabolic cost of walking and compared it to walking without assistance and walking without the exoskeleton. We measured changes in kinematics and muscle activity to see how users adapted to assistance and to gain insights into the potential biomechanical mechanisms that brought about reductions in metabolic cost. By finding and comparing optimized assistance for different potential device architectures, we expect these results to inform models of human adaptation to exoskeletons and lead to the design of more effective exoskeletons.

## Methods

### Participants

Four healthy participants were included in this study (P1: M, 26 years old, 90 kg, 187 cm; P2: F, 26 years old, 61 kg, 170 cm; P3: M, 19 years old, 82 kg, 176 cm; P4: M, 23 years old, 62 kg, 171 cm). We were limited to four participants because of the extensive time required to complete the protocol (Supplementary Material, Sections 11 and 12). Each participant completed at least 50 hr of experiments in total. These participants were also authors of the study (P.W.F., G.M.B., R.M.M., and R.R.) as these were the people who could spend such time as a participant for the study. All four participants completed the whole-leg optimization, but due to external factors related to the COVID-19 pandemic, three participants completed one-joint (P1, P2, and P3) and two-joint (P1, P3, and P4) optimization. With three participants, we have a statistical power of 0.8 to detect metabolic reductions greater than 24%, assuming metabolic reductions have a standard deviation of 7.4% (Zhang et al., 2017; Supplementary Material, Section 12). With four participants, the 50% reduction detected from whole-leg assistance has a statistical power of 0.999.

All four participants were experienced with the device at the time of optimization. P1 and P2 had previous experience walking in the exoskeleton before this experiment. P3 and P4 completed a training protocol prior to optimization to get accustomed to wearing the exoskeleton and walking with torques. More details on the training protocol are included in Supplementary Material, Section 1.

### Experimental Protocol

**Figure 1.**
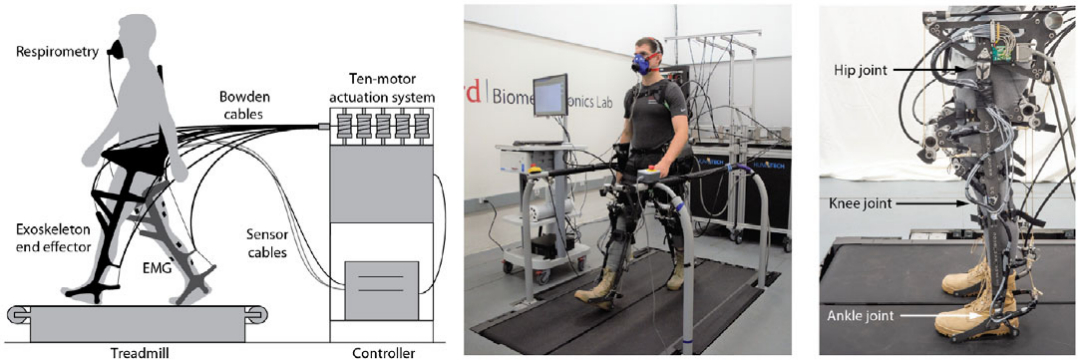
Overview of exoskeleton emulator system. (Left) Overview of exoskeleton emulator. Ten powerful off-board motors actuate a lightweight end effector worn by a user who walks on a treadmill. Metabolic cost is measured using a respirometry system and muscle activity is measured using electromyography (EMG). (Center) Isometric photo of experimental setup. (Right) Side view of exoskeleton. The exoskeleton can apply torques in hip flexion and extension, knee flexion and extension, and ankle plantarflexion.

We optimized single-joint, two-joint, and whole-leg assistance using a hip–knee–ankle exoskeleton (Figure 1; Bryan et al., 2020). We used human-in-the-loop optimization (Zhang et al., 2017), a strategy where the control of the exoskeleton is updated in real-time based on measurements of the user. For this study, the cost function to be minimized was the measured metabolic cost of walking at 1.25 m/s. First, we optimized single-joint assistance for P1, P2, and P3 in the order of ankle-only, hip-only, and then knee-only assistance. We then optimized whole-leg assistance for all four users, meaning we optimized assistance of the hip, knee and ankle simultaneously. Finally, for P1, P3, and P4, we optimized two-joint assistance, in the order of hip–ankle, knee–ankle, and hip–knee assistance.

After optimization, we performed validation experiments to compare the optimized assistance to the control conditions of walking without the exoskeleton and walking in the exoskeleton with no torque applied. During these validations, we measured metabolic cost, applied torques, kinematics, and muscle activity.

### Exoskeleton Hardware

Assistance was applied using a hip–knee–ankle exoskeleton emulator (Figure 1; Bryan et al., 2020). This device can apply large torques using offboard motors and Bowden cable transmissions to actuate an end effector worn by the user, enabling laboratory tests of different assistance strategies without actuation limits (Caputo and Collins, 2014). The device has a worn mass of 13.5 kg. It has carbon fiber struts along the length of the legs that are designed to minimize restriction of the user by being stiff in actuated directions but compliant in out-of-plane bending. The exoskeleton was fit to each user by adding boots for their foot size, by adjusting the length of the shank, thigh, and torso segments of the exoskeleton, and by adjusting the width of the exoskeleton at the knees, thighs, and hips. Straps were adjusted to fit the user at the shanks, thighs, hips, and torso.

### Exoskeleton Control

The exoskeleton is controlled by commanding a desired torque for each joint (Bryan et al., 2020). When the desired torque is zero, the exoskeleton tracks the user’s joint angles and applies no torques. During walking, we define these desired torque profiles as a function of percent stride. We consider heel strike, measured by ground reaction forces on the treadmill, to be the start of a stride. We calculate percent stride as the time since heel strike divided by the average stride time over the past 20 strides. The hip profile starts at 84% of stride after heel strike because hip extension torque is active during heel strike. Resetting the hips’ stride time at heel strike caused discrete jumps in hip extension torque during pilot testing. The desired torque profile for each joint is made up of a spline (piecewise cubic hermite interpolating polynomial) anchored by nodes. Each node can be set in advance by an operator, or it can be updated in real time by an algorithm. For the knees, torque was also commanded as a function of joint state. Along with a torque-time profile, knee torque had one spring-like phase during stance, and one damping-like phase during late swing.

Our exoskeleton accurately applied desired torques using closed-loop proportional control with iterative learning and joint-velocity compensation (Zhang et al., 2015; Bryan et al., 2020). Root-mean-square (RMS) error for tracking desired torques was 0.6 Nm at the hips, 3.0 Nm at the knees, and 0.4 Nm at the ankles during whole-leg assistance (Supplementary Material, Section 13). Error was highest at the knees because the state-based control allowed for discontinuous jumps in desired torque that were not possible for our device to track, and the desired torque would change step to step making it harder to track. When zero torque was commanded it was realized effectively, with an RMS applied torque of less than 1 Nm.

### Controller Parameterization

**Figure 2.**
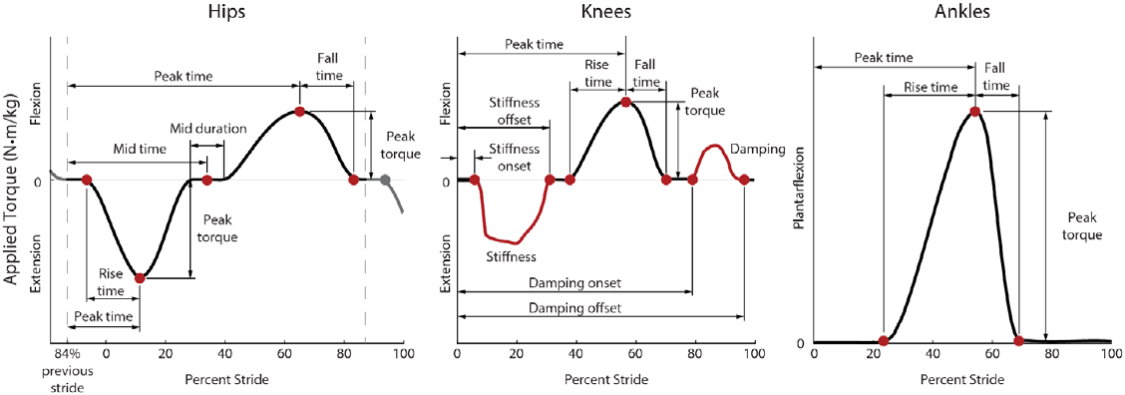
Desired torque profiles defined by the controller for the hips, knees, and ankles. Hip-only assistance was defined by 8 parameters, knee-only assistance was defined by 10 parameters, and ankle-only assistance was defined by 4 parameters, meaning whole-leg assistance was optimized using 22 parameters. For the hips (left) and ankles (right), torque (black) was commanded as a function of time, defined as a spline fit to nodes (red) that were optimized during the experiment. For the knees (center), torque was commanded both as a function of time (black), joint angle, and joint velocity. During stance, the knee torque was a function of knee angle to mimic a spring (red), where the spring’s stiffness was optimized. During late swing, torque was a function of knee joint velocity to mimic a damper (red). The red curves shown for these periods of state-based control are the average applied torque at the knees from whole-leg assistance, but the applied torque could vary based on the user’s kinematics. The steep increases and decreases in applied torque during knee extension assistance were due to the impedance controller being turned on and off as a function of percent stride defined by the nodes while the knee angle was nonzero, resulting in discrete jumps in desired torque.

The optimization algorithm varied parameters that affected the desired torque control of the exoskeleton (Figure 2). These parameters are mostly related to the timing and torque magnitude of the nodes that define our splines.

We chose these parameters by considering previously successful human-in-the-loop optimizations (Zhang et al., 2017; Ding et al., 2018), considering biological torques during walking (Winter, 1991), and by pilot testing. Before this, one participant completed a 9-parameter whole-leg optimization pilot study (Supplementary Material, Section 14), which indicated the need for more degrees of freedom in our controller.

For these optimizations, we included 8 parameters for the hips, 10 for the knees, and 4 for the ankles (Figure 2). For optimization of whole-leg assistance, the optimizer could adjust all 22 parameters. Each parameter had a minimum and maximum allowed value. The allowed parameter ranges were based on user testing to ensure all tested profiles would be sufficiently comfortable for the user to walk in. The optimization tended not to optimize to these limits. However, ankle torque was often as large and as late in stride as possible, which meant the fall time was minimized to prevent torque application during swing. During single-joint assistance, ankle torque magnitude was limited to 1 Nm/kg. After single-joint optimization concluded and multi-joint optimization began, participants noted that these torques were too large to walk in comfortably. Participants noted that the ankle assistance felt like it was extending the ankle too quickly, and it is possible that kinematic adaptations during ankle-only assistance that mitigated this discomfort were not possible when assistance was also present at the hip and knee. To prevent discomfort, the torque magnitude limit was reduced to 0.8 Nm/kg for ankle torque during multi-joint assistance. Tables with the parameter ranges, as well as their initial and optimized values, are available in Supplementary Material, Section 5.

The hip profile was defined by eight parameters. Hip extension was defined by the rise time, peak time and peak magnitude while hip flexion was defined by the peak time, peak magnitude and fall time. There was a period of no torque in between the two peaks defined by the midpoint timing and its duration. The period of no torque dictated the hip extension fall time and the hip flexion rise time.

Knee torque was defined by 10 parameters. Knee torque was commanded both as a function of percent stride and of joint state. The first phase of knee torque was knee extension defined as a virtual spring, with torque proportional to knee angle, which was zero when the knee was straight. Knee extension was defined by the virtual spring onset timing, stiffness, and offset timing. If the joint angle reached zero degrees before the offset time, the knee torque would stay at zero torque for the remainder of the stiffness period. During knee flexion around toe-off, torque was defined as a function of time similar to the hips. This torque was determined by the rise time, peak torque magnitude, peak time, and fall time. Late in swing, knee flexion torque was commanded as a virtual damper, so torque was proportional to a filtered measurement of knee joint velocity. The damping period was defined in a similar way to knee stiffness, with optimization of the onset timing, the damping coefficient, and the offset timing. As the knee joint angle and velocity were not necessarily zero at the start of the state-based controllers, desired torque could instantaneously change at the onset.

Ankle torque was defined using four parameters, which were previously effective for optimization of ankle assistance (Zhang et al., 2017). Torque was defined by rise time, peak torque magnitude, peak time, and fall time. To ensure large torques were not applied too late in the stride, torque was set to be zero by 65% of stride, so if peak time optimized to its latest allowed value (55% of stride), the fall time would be set to the minimum allowed fall time (10% of stride).

### Human-in-the-Loop Optimization Protocol

To optimize assistance, we used the covariance matrix adaptation evolutionary strategy (CMA-ES) (Hansen, 2006), which has previously been effective for human-in-the-loop optimization of exoskeletons (Zhang et al., 2017; Witte et al., 2020). CMA-ES samples a “generation” of conditions from a distribution defined by parameter means and a covariance matrix, ranks the performance of the samples, and uses those results to update the mean and covariance before sampling the next generation. The optimizer’s goal was to minimize metabolic cost, which was estimated for each condition after 2 min of walking using a first-order dynamical model (Selinger and Donelan, 2014), similar to previous work (Zhang et al., 2017; Witte et al., 2020). More details about the optimization, including hyperparameters and numbers of conditions per generation, are included in Supplementary Material, Section 15.

The initial parameter values for each optimization were carefully selected to try to reduce convergence time. For the single-joint optimizations for P1, initial parameter values were based on previously optimized assistance (Zhang et al., 2017; Ding et al., 2018), hand-tuning, and a 9-parameter pilot study (Supplementary Material, Section 14). For whole-leg optimization for P1, initial values were based on the optimized values for single-joint assistance. For P2, P3, and P4, initial values for optimization were based on the optimized values for P1. Finally, for the two-joint assistance optimizations, initial parameters were based on each participant’s previously optimized whole-leg assistance values. The initial values for all parameters are included in Supplementary Material, Section 5.

The optimization time was intended to balance being long enough to ensure convergence while short enough to be experimentally feasible. P1 underwent a longer optimization to ensure convergence, to estimate expected reductions, and to inform our understanding of how the optimizer would perform (Supplementary Material, Sections 11 and 16). Ankle-only optimization was conducted for 12 generations over 3 days, and hip-only, knee-only and whole-leg were conducted for at least nine generations over 3 days. Each two-joint assistance optimization was conducted for six generations over 2 days. For all participants, single-joint and whole-leg optimization each occurred over at least 3 days, which seemed sufficiently long for P1 to reach metabolic reductions that were consistent in future sessions and that matched previous studies for previously assisted joints (Zhang et al., 2017) (Supplementary Material, Section 11). For whole-leg assistance for P3, the optimization was restarted because the user had an abnormally high stride frequency that had high metabolic cost, indicating a maladaptation to assistance similar to some users in a previous optimization study. The exact number of generations and days for each optimization is included in Supplementary Material, Section 11. Participants were permitted but not required to take breaks between generations. While walking, participants were allowed to listen to podcasts using wireless headphones.

### Validation Protocol

We conducted validation experiments to evaluate the effectiveness of the optimized assistance. Metabolic reductions were validated for each assistance strategy after each optimization. After all the single-joint and whole-leg optimizations were completed, torques, kinematics, and muscle activity were compared between assistance strategies on the same day. Finally, the two-joint assistance strategies were validated after each optimization.

After each optimization was completed, a validation experiment was used to accurately assess the metabolic cost of walking and calculate the percent reduction. This collection was on a separate day, before optimization of the next assistance strategy began. Users walked in longer bouts for the exoskeleton conditions to ensure accurate measurements of steady-state metabolics and to ensure users were acclimated to the device and assistance. We recorded the user standing quietly for 6 min, walking without the exoskeleton for 6 min, walking in the exoskeleton with no torque applied for 10 min, and walking with the optimized assistance torques for 20 min, in a double-reversed order (ABCDDCBA). This order was not randomized due to the time it takes to get in and out of the exoskeleton, as well as to maximize acclimation to the device by presenting progressively more novel conditions. Users rested for at least 3 min between walking conditions, and at least 5 min before a quiet standing condition, to ensure their metabolics returned to baseline. For the no exoskeleton condition, users wore the same brand and model of boots that are included in the exoskeleton (McRae 8189).

After all single-joint and whole-leg optimizations were completed, we evaluated all these optimized strategies in one data collection to directly compare conditions. We measured applied torque, kinematics, muscle activity, power, and vertical ground reaction forces. For this validation, we recorded the user standing quietly for 6 min, walking without the exoskeleton for 6 min, walking in the exoskeleton with no torque applied for 10 min, and then walking for 10 min in each of the four optimized assistance conditions (hip-only, knee-only, ankle-only, and whole-leg) in a random order, and then the no torque, no exoskeleton, and quiet standing conditions a second time (ABCDEFGCBA).

For the three users who completed optimization of two-joint assistance, a validation day was completed after each optimization similar to the protocol following the single-joint and whole-leg optimizations. We recorded the user standing quietly for 6 min, walking without the exoskeleton for 6 min, walking in the exoskeleton with no torque applied for 10 min, and walking with the optimized assistance torques for 20 min, in a double-reversed order (ABCDDCBA), so each condition was evaluated twice. For these validations, we measured metabolics, torques, kinematics, muscle activity, power, and vertical ground reaction forces.

### Measured Outcomes

We collected biomechanical data of the user when walking in the different conditions during the validation. We calculated the average of these measurements over the last 3–5 min of walking of each condition to ensure the user’s metabolics and gait had reached steady-state.

#### Metabolic cost

Metabolic cost was calculated using indirect calorimetry. We measured volumetric carbon dioxide expulsion, oxygen consumption, and breath duration on a breath-by-breath basis (Quark CPET, COSMED). For each condition, we calculated metabolic rate using a modified Brockway equation (Brockway, 1987) similar to previous studies (Zhang et al., 2017; Witte et al., 2020). Average metabolic cost was calculated for each condition using the last 3 min for quiet standing and walking with no exoskeleton, and using the last 5 min for walking with no torque and walking with optimized assistance. Because each condition was evaluated twice in the double-reversed order, the average was calculated across both measurements of the condition. The cost of quiet standing was subtracted from the measured cost of all the other conditions to calculate the cost of walking. Users fasted for at least 2 hr before optimizations and at least 4 hr before validations to minimize the possible thermal effects of food on metabolic cost measurements. Two-tailed paired *t*-tests were used to evaluate if the metabolic cost of walking with exoskeleton assistance was significantly different from walking in the control conditions.

For all two-joint optimizations except P3 hip-ankle, participants wore a cloth mask underneath the metabolics mask to comply with COVID-19 safety protocols. This mask did affect the metabolics measurements, by seeming to cause an underestimation of measured metabolic cost (Supplementary Material, Section 17). While this disrupted the accuracy of the absolute measurements, we expected the percent reductions in metabolic cost to be accurate, because we are comparing between conditions. We excluded these affected measures when calculating the average absolute measurements reported for standing quietly, walking with no exoskeleton, and walking with no torque.

#### Torques and kinematics

Applied torques were measured using load cells and strain gauges on the exoskeleton. Exoskeleton joint angles were recorded to estimate user kinematics, meaning that we could not calculate kinematics for walking without the exoskeleton. Stride frequency was calculated using vertical ground reaction forces measured by the instrumented treadmill (Bertec). Measurements were averaged over the last 3 min of the condition for walking with no exoskeleton, and averaged over the last 5 min for walking with no torque and walking with each assistance condition. Measurements were averaged across both legs. For conditions that were evaluated twice (walking with no exoskeleton and walking with no torque), results were averaged across the two conditions.

Biological torques reported for reference (Figure 4) were from a separate study. Reference walking data is from three able-bodied male subjects (Arnold et al., 2013), different from the participants included in this study. Walking data was collected from three gait cycles of motion capture data during treadmill walking at 1.25 m/s including marker trajectories, ground reaction forces, and EMG measurements (Arnold et al., 2013). Biological joint torques during walking were calculated using the Inverse Dynamics tool in OpenSim (Seth et al., 2018), as described in Franks et al. (2020).

#### Muscle activity

Muscle activity was measured using surface EMG (Delsys Trigno). We applied a third-order bandpass filter of 40–450 Hz, rectified, then applied a third-order low pass filter of 10 Hz (De Luca et al., 2010). Muscle activity was averaged for a stride over the last 5 min of the device conditions and over the last 3 min for the no device conditions. We subtracted the baseline noise offset then normalized to the maximum of the no torque condition profile. The sensor locations are similar to the protocol of previous gait analysis experiments (Winter, 1991) with adjustments to avoid interfering with the device structure and straps.

## Results

### Metabolic Cost

**Figure 3.**
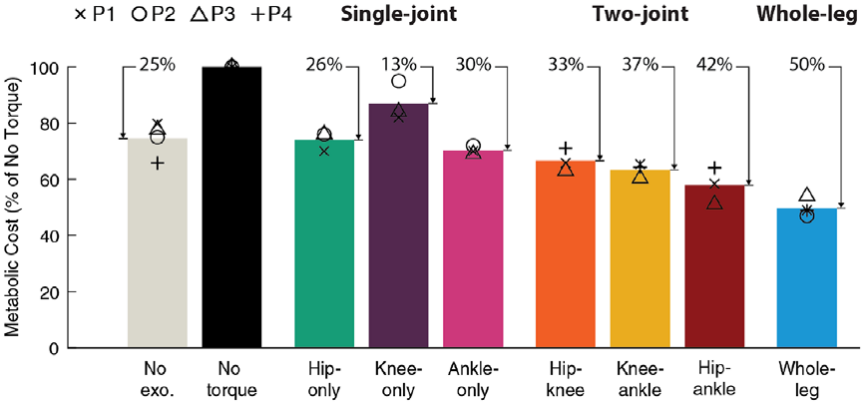
Metabolic cost of walking. Average metabolic cost (bar) of each condition reported as a percentage of walking in the exoskeleton with no torque. Individual participant values are shown with symbols (P1 X, P2 O, P3 Δ, and P4 +). Metabolic cost of walking was calculated by subtracting out quiet standing. The percent reduction relative to walking with no torque is shown above each bar. For each participant, the cost of walking without the exoskeleton (No exo., gray) was averaged over all validations. Whole-leg assistance (blue, *N* = 4) provided the largest improvement to metabolic cost of walking, reducing it by 50% relative to walking in the exoskeleton without assistance.

The metabolic cost of walking was reduced in all assistance conditions (Figure 3). To evaluate the metabolic cost of walking, we subtracted the metabolic cost of standing quietly (1.52 W/kg on average, Supplementary Material, Section 2) from the metabolic cost measured in each walking trial. The metabolic cost of walking without wearing the exoskeleton was 2.86 W/kg on average. The cost of walking in the exoskeleton with no torque applied was 3.92 W/kg on average, which was higher than the no-exoskeleton condition because of the added mass and impedance to the user. Percent reductions in metabolic cost were calculated in comparison to this no-torque condition to assess the effect of the designed torque assistance specifically without the effect of device mass, allowing for best comparisons between assistance conditions, which could require different device architectures as mobile systems.

Single-joint assistance at each joint reduced metabolic cost, with the largest improvements coming from the ankles and hips. Hip-only assistance reduced the metabolic cost of walking by 26% relative to walking with no torque (*N* = 3, range of reductions: 24–30%, *p* = .005). Knee-only assistance reduced metabolic cost for each participant, with an average reduction of 13% relative to walking with no torque, although this was not statistically significant (*N* = 3, range of reductions: 5–18%, *p* = .07). Ankle-only assistance performed best of the single-joint strategies, reducing metabolic cost by 30% relative to walking with no torque (*N* = 3, range of reductions: 28–31%, *p* = .004). When assisting a single joint, exoskeleton designers should consider the ankles or the hips.

Two-joint assistance outperformed single-joint assistance. Two-joint assistance reduced metabolic cost of walking relative to no torque by 33% for hip–knee (*N* = 3, range of reductions 29–37%, *p* = .008), 37% for knee–ankle (*N* = 3, range of reductions 35–40%, *p* = .02), and 42% for hip–ankle assistance (*N* = 3, range of reductions: 36–49%, *p* = .03) (Supplementary Material, Section 3). Hip–ankle assistance provided the most benefit, mirroring single-joint reductions and aligning with expectations based on biological power.

Whole-leg exoskeleton assistance led to the greatest reductions in metabolic cost of any condition. Whole-leg assistance (hips, knees, and ankles simultaneously) reduced the metabolic cost of walking by 50% relative to walking with no torque (*N* = 4, range of reductions: 46–53%, *p* = .003), corresponding to a reduction of 33% relative to walking without wearing the exoskeleton (*N* = 4, range of reductions: 17–41%, *p* = .016). This shows that about half of the metabolic energy expended during walking can be saved through exoskeleton assistance, and suggests that whole-leg assistance could provide large net benefits in untethered systems, even after accounting for the effects of added mass.

### Optimized Exoskeleton Torque

**Figure 4.**
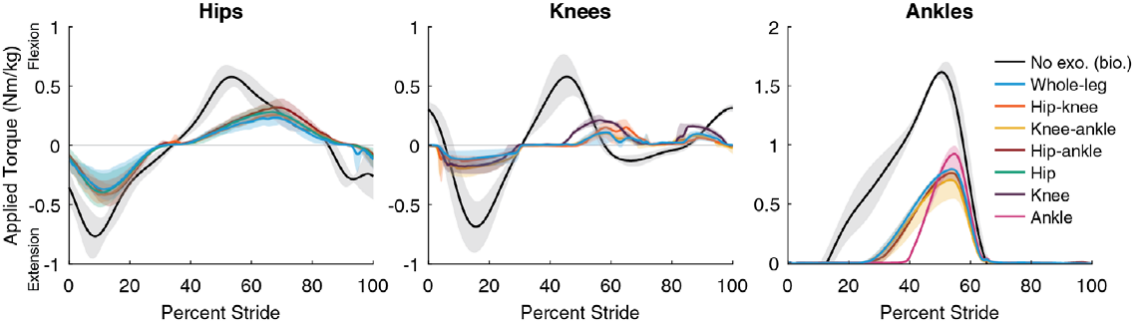
Optimized exoskeleton torques. Optimized single-joint (green, purple, and pink), two-joint (orange, yellow, and red) and whole-leg (blue) exoskeleton assistance torques at the hips (left), knees (center), and ankles (right). Lines are the average of the measured applied torque profiles across both legs and all participants (*N* = 3 for single-joint and two-joint, *N* = 4 for whole-leg), with the range of optimized profiles shown with their respective clouds for each type of assistance. Biological joint torques for unassisted walking without an exoskeleton (black) are included from a different study with different participants (Arnold et al., 2013; Franks et al., 2020) for reference; gray clouds indicate standard deviation of biological torques. For the hips and knees, whole-leg assistance optimized to smaller magnitudes than single-joint assistance. For the ankles, maximum torque had to be constrained to find comfortable profiles for walking. Ankle torques were limited to 1 Nm/kg for single-joint assistance, and 0.8 Nm/kg for two-joint and whole-leg assistance.

Optimized torques differed from biological torques in both timing and magnitude (Figure 4, Supplementary Material, Sections 4–6). Optimized torque magnitudes were smaller than biological torques, with peak exoskeleton torques ranging from about 15 to 60% of biological peaks. Ankle torque magnitudes were largest and optimized to the comfort-limited parameter constraints in all but two cases. The timing of the optimized assistance only partially aligned with biological torque. For example, the peak of optimal hip flexion assistance occurred later than peak biological flexion torque. Sometimes, assistance torque opposed typical biological torques. For example, knee flexion assistance around 60% of stride opposed biological knee extension torque for normal walking. These optimized magnitudes indicate the design requirements for mobile devices and show that optimized assistance is not a scaled version of biological torques.

The shape and timing of optimized assistance was consistent across conditions and participants, but optimal magnitudes differed. For example, the optimal timing of peak hip extension assistance was about 11% of stride for all joint combinations and participants, while optimal magnitudes ranged from 0.24 Nm/kg (hip-only) to 0.5 Nm/kg (hip–knee). One exception to the consistency of optimal timing was ankle torque rise time, which was shorter during single-joint assistance, possibly due to adjustments for comfort in multi-joint conditions (Supplementary Material, Section 5). Optimized assistance torques were typically larger when acting alone at a joint, and smaller when acting in a multi-joint configuration. For example, for P1, applied knee flexion torque peaked at 0.25 Nm/kg for knee-only assistance and at 0.14 Nm/kg for whole-leg assistance. The consistency of optimal timing parameters suggests that optimization could occur in a lower-dimensional parameter space of torque magnitudes, and that a generalized assistance profile could be almost as effective as a customized one.

### Kinematics

**Figure 5.**
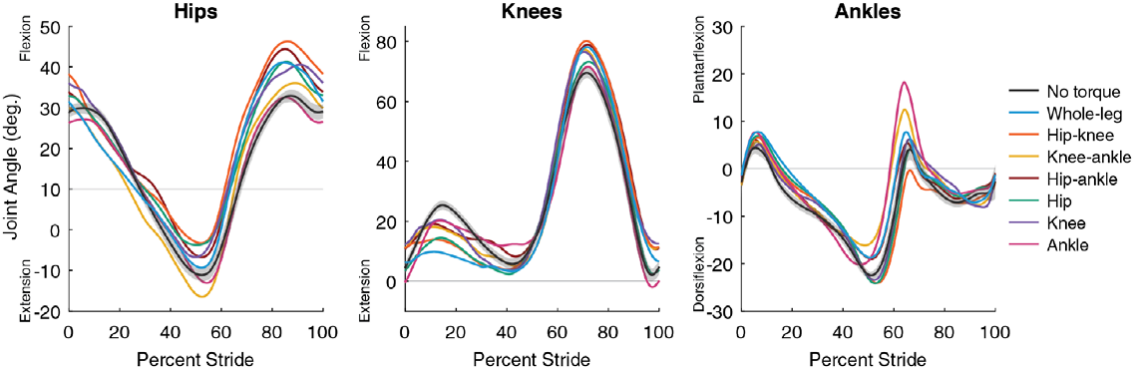
Average joint kinematics. Average joint angle as a percentage of stride at the hips (left), knees (center), and ankles (right) for each assistance condition (denoted by color). Shown here are the average for both legs across all participants (*N* = 3 for single-joint and two-joint, *N* = 4 for whole-leg). All single-joint and whole-leg conditions for P1, P2, and P3 were tested on the same day to reduce changes in alignment between user and device. Two-joint and P4’s three-joint conditions were each collected individually. For walking in the exoskeleton with no torque (black), the standard deviation of angles is shown (gray cloud) to contextualize the magnitude of changes between conditions.

Kinematics varied between assistance conditions, indicating that the user’s walking pattern is not fixed and adapts to best utilize assistance (Figure 5, Supplementary Material, Sections 7 and 8). These changes were beyond the deviation measured during walking with no torque (gray cloud, Figure 5). In some cases, assistance shifted joint angles in the direction of the applied torque. For example, peak ankle plantarflexion angle increased with whole-leg assistance, and increased even more during ankle-only assistance, which had larger ankle torques. However, some kinematic changes were not the direct result of applied torques. For example, the indirect effects of hip-only and ankle-only assistance on the knee during stance were larger than the direct effect of knee assistance. These kinematic adaptations indicate the user adjusts their walking strategy to maximize the benefit they get from the exoskeleton, and that these adaptations do not always match intuition.

### Muscle Activity

**Figure 6.**
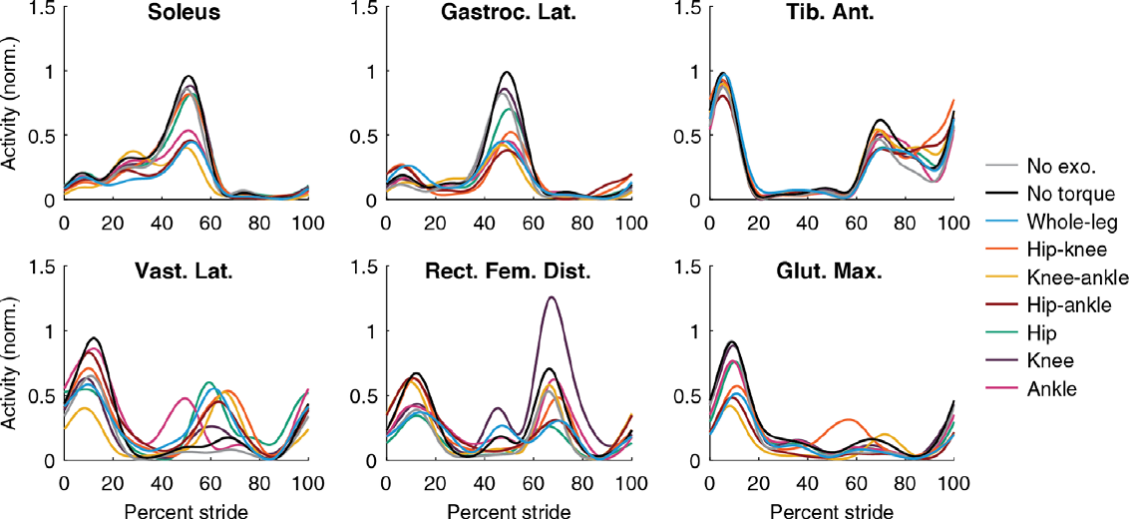
Muscle activity. Muscle activity measured during walking using surface EMG for each condition. Lines shown are the average across all participants (*N* = 3 for single-joint, hip–knee, and hip–ankle, *N* = 2 for knee–ankle due to a technical difficulty with P3’s EMG collection, and *N* = 4 for whole-leg). The EMG signal was filtered, averaged, had baseline activity removed to eliminate noise, and normalized to the peak value of walking in the exoskeleton without assistance (black). Gluteus maximus activity (second row, third column) decreased for hip-only, hip–knee, hip–ankle, and whole-leg assistance as expected, and also decreased during ankle-only and knee–ankle assistance, indicating that the gluteus was indirectly assisted by ankle exoskeleton torque. This effect was less pronounced for the soleus (top row, first column), where hip-only and hip–knee assistance only slightly reduced muscle activity.

Muscle activity decreased with assistance, but it was not completely eliminated (Figure 6; Supplementary Material, Section 9). Typically, reductions in activity were seen in muscles that crossed assisted joints and acted in the same direction as assistance. For example, soleus activity decreased during all conditions that applied ankle assistance. Sometimes activity increased during opposing assistance, such as in the vastus lateralis during periods of knee flexion torque. Some reductions in muscle activity occurred during assistance at other joints. For example, gluteus maximus activity decreased when the hip was assisted directly, but also decreased during ankle-only assistance. This is consistent with the observation that exoskeleton assistance at one joint can indirectly assist muscles that cross other joints. The indirect assistance could be from the complex dynamics of the leg during walking, or could be facilitated by the kinematic adaptations to maximize the effectiveness of each type of assistance. Optimized assistance did not cause users to eliminate muscle activity, suggesting that either some amount of activity is still useful or further advancements to control architecture would be needed to reduce energy expenditure further.

## Discussion

With capable devices, optimization, and training, exoskeletons can provide very large improvements in locomotor performance. Whole-leg assistance reduced the metabolic cost of walking by 50% relative to walking with no torque, a substantial improvement over the state-of-the-art (17–24%) (Zhang et al., 2017; Ding et al., 2018; Lim et al., 2019). This corresponded to a 33% reduction relative to walking with no exoskeleton, much greater than the just-noticeable difference in metabolic cost (20%) (Medrano et al., 2020), indicating that participants could feel the reduction in effort compared to walking normally. Because whole-leg assistance produced the largest benefit of all assistance conditions, exoskeleton designers who want to maximize performance should consider assisting the whole leg.

Among single-joint assistance strategies, ankle-only assistance was most effective, followed closely by the hip, with smaller reductions possible at the knee. Hip and knee assistance resulted in greater metabolic cost reductions than previous lower-torque exoskeletons assisting these joints (Ding et al., 2018; Lim et al., 2019; MacLean and Ferris, 2019), while ankle assistance resulted in similar improvements as found with high-torque exoskeletons and human-in-the-loop optimization (Zhang et al., 2017; Jackson and Collins, 2019). Knee-only assistance reduced the metabolic cost of walking, although the reduction was not statistically significant for our sample size and significance level. Knee-only assistance may be more effective for walking up inclines (MacLean and Ferris, 2019; Haufe et al., 2020), considering the increased positive power requirements from the knee (Montgomery and Grabowski, 2018). Devices designed to assist just one joint during level walking should target the ankle or hip, which showed reductions of 30 and 26%, respectively. With similar metabolic reductions, designers could compare between the ankle or hip based on other aspects of the design. For example, it may be easier to interface with the ankle using a simple device, while a hip exoskeleton places the mass more proximally on the body where it is easier to carry (Browning et al., 2007).

The best-performing two-joint assistance strategy, hip-ankle assistance, reduced metabolic cost by 42%, nearly as much as whole-leg assistance. With 37% and 33% reductions for knee–ankle and hip–knee assistance, the small added benefit of knee-assistance may not be worth the added device complexity compared to ankle-only or hip-only assistance for level ground walking. The inclusion of knee assistance may be more effective for different walking conditions such as incline walking or during sit-to-stand, where the knee is expected to contribute more to movement.

Assisting multiple joints results in larger net benefits, but smaller benefits per joint, possibly because of the way people adapt to exoskeleton assistance. Whole-leg assistance led to the largest metabolic cost reduction (50%), but it was smaller than the sum of the reductions of the single-joint assistance strategies (26% + 13% + 30% = 69%). During single-joint assistance, users may have been able to adapt the kinematics of their unassisted joints to take most advantage of the single-joint assistance. During whole-leg assistance, their subconscious walking strategy could have been to maximize metabolic benefit overall from assistance of the whole-leg, while not getting maximal benefit from any joint in particular. This adaptation might also explain why optimized torque magnitudes during single-joint assistance were larger than whole-leg torque magnitudes. It was previously hypothesized that multi-joint assistance may be more effective at assisting biarticular muscles, but the relative benefit of single-joint assistance indicates that indirectly assisting muscles at other joints from single-joint assistance leads to larger benefits on a per-assisted-joint basis. The reduction in metabolic cost per joint assisted (Table 1) could be helpful for designers when considering potential device architectures.

**Table 1.** Metabolic reduction per joint assisted, relative to walking in the exoskeleton with no torque

Condition	Ankle-only (%)	Hip-only (%)	Hip–ankle (%)	Knee–ankle (%)	Whole-leg (%)	Hip–knee (%)	Knee-only (%)
Reduction per joint	30	26	21	18	17	16	13
Total reduction	30	26	42	37	50	33	13

These results suggest ways of designing better exoskeleton products. Optimized torques did not mimic biological torques, with magnitudes smaller than biological for all joints and peak torques later than biological peaks for the hips and ankles (Figure 4). Optimized torque magnitudes were within the range of reported capabilities of some existing mobile devices for the lightest participant (61 kg) (Mooney et al., 2014; Shepherd and Rouse, 2017; Lee et al., 2018) but not for the heaviest participant (90 kg) (Bryan et al., 2020). Exoskeletons could be designed in different sizes (e.g., small, medium, and large) that meet the optimized torque magnitudes for different sized users while minimizing worn mass. The similar timing parameters across users and across assistance strategies suggest that these optimized profiles could translate well to existing devices with lower capabilities and could be generalizable to a wide range of users. Device designers could consider control strategies that allow for kinematic adaptations because they seem to be useful to maximize device effectiveness. We also recommend considering state-based control at the knees, which was more effective than pilot tests of strictly torque-time control. This strategy may have facilitated adaptation to knee extension assistance during stance, because the torques grew as the user bent more into flexion, allowing for a “stabilizing” effect that prevented buckling of the knee. In this study, we used a tethered exoskeleton emulator to compare assistance, but future work should attempt to recreate this assistance on mobile exoskeletons. We reported our improvements primarily relative to walking in the exoskeleton unassisted, and we expect these relative improvements to be consistent for mobile systems, but future mobile device development will need to focus on keeping worn mass low to ensure a good benefit relative to no exoskeleton as well. These findings should translate well to a whole-leg mobile device because the worn mass (13.5 kg) is similar to the expected mass of a mobile device capable of the optimized torque magnitudes (10 kg) based on published torque densities (Pratt et al., 2004; Zoss et al., 2005; Meijneke et al., 2014; Mooney et al., 2014; Giovacchini et al., 2015; Seo et al., 2016; John et al., 2017; Shepherd and Rouse, 2017; Bryan et al., 2020; Supplementary Material, Section 10).

These results can be used to improve our models of human coordination, especially when using assistive devices. The larger metabolic cost reductions we saw from hip and ankle assistance support the idea that the hips and ankles are primary energy consumers during walking (Farris and Sawicki, 2012). Unlike some simulations (Franks et al., 2020), muscle activity did not go to zero even when assisted without hitting torque limits, indicating the user is optimizing for more than just metabolic cost for an average steady-state stride. Simulations could capture that more complicated objective function, including control required for balance. These results show that kinematic adaptations to assistance are important and should be considered in simulations. Biomechanical models could also be used to study the kinematic adaptations and relative metabolic benefits seen in this study. Muscle-level simulations of single-joint and multi-joint assistance could better understand how assistance at one joint could be indirectly assisting muscles at another joint, facilitated by the user’s kinematic adaptations. This work could also try to understand how single-joint and multi-joint assistance compare in assisting biarticular muscles specifically.

This study could have been improved by testing more participants, providing additional training, or testing additional controller parameterizations. This was an extremely arduous experiment with long optimization times, with each participant having completed over 50 hr of experiments. It was then interrupted by difficult external conditions (the COVID-19 pandemic). As such, we were only able to complete three participants for the single-joint and two-joint optimizations, and four participants for the whole-leg optimizations. However, given the magnitude of the changes and the consistency of the responses across participants, this sample size is sufficient to identify the efficacy of the joint combinations tested. Given three participants and a desired statistical power of 0.8, and assuming metabolic reductions have a standard deviation of 7.3% (Zhang et al., 2017), we can confidently detect metabolic reductions of 24% and larger. Although we gave our users substantial training and optimization time, more time may have improved the outcomes. Longitudinal studies with mobile devices that can be worn daily could show greater improvements to walking as users adapt. During optimization, torque magnitudes reached the comfort-based limits at the ankles, which were set due to discomfort at the biological ankle joint, possibly from extending the ankles too quickly or too far during torque application at push-off. If we were able to apply larger torque magnitudes comfortably, we could unconstrain the optimization of assistance and allow the torques to converge on the magnitude that provides the greatest benefit, which could be larger than 1 Nm/kg for the ankles. Using a more sophisticated control approach to ensure user comfort while allowing the largest possible exoskeleton torques might also lead to larger benefits. Due to the COVID-19 pandemic interrupting our protocol, participants had to wear cloth masks during optimization and validation of two-joint assistance, which could have impacted the accuracy of the metabolic cost measurements. While we believe the percent reductions should still be accurate, future work studying two-joint exoskeleton assistance could confirm the results found here.

These results suggest that new cost functions, gait environments, and user populations could be exciting topics for future studies. Future work could optimize metabolic cost alongside other costs that are important for gait, such as walking speed, balance, or user satisfaction (Ingraham et al., 2020). In our study, we did not rigorously collect user feedback, but participants often provided similar feedback, such as noting that the exoskeleton felt like it was making them march if torques were too large, or that they noticed how beneficial assistance was right after it was turned off. Future work could collect user feedback in an organized way and incorporate it in the design of exoskeleton assistance, possibly through human-in-the-loop optimization. Our study did not penalize high torques or powers, but future work could try to maintain sufficient metabolic cost reductions while minimizing actuator requirements, which can be costly to mobile devices. While our study assisted walking at a fixed speed on level-ground, future work can explore optimized assistance for walking in different conditions such as at different speeds, on inclines, or with worn loads. Our study was restricted to a treadmill due to our tethered device, but this work could be extended to unstructured environments by translating the paradigm to mobile devices. Our findings for assisting young, able-bodied users could be a starting point to optimize assistance for older adults and people with disabilities, hopefully speeding the discovery of effective assistance strategies.

## Data Availability

The data that support the findings of this study are available on request from the corresponding author, P.W.F., and will be made available online at biomechatronics.stanford.edu following publication.

## Supplementary material

For supplementary material accompanying this paper visit https://doi.org/10.1017/wtc.2021.14. click here to view supplementary material
