# Supplementary material for: Comparing optimized exoskeleton assistance of the hip, knee, and ankle in single and multi-joint configurations
Source: Wearable Technol. 2021 Nov 24;2:e16. doi: 10.1017/wtc.2021.14 (PMC10936256; doi:10.1017/wtc.2021.14)
Supplement: Supplementary file 1 [file wtcsup.zip › S2631717621000141sup001.pdf]

**Supplementary table of contents**

1. User training
2. Metabolics - single-joint and whole-leg
3. Metabolics - two-joint
4. Applied torques for each participant
5. Optimized parameters and parameter ranges
6. Applied exoskeleton power
7. Kinematics for each participant
8. Stride frequency for each participant
9. Muscle activity for each participant
10. Mobile device mass estimate
11. Experiment log - generations and reductions per day
12. Sample size and power analysis
13. Torque-tracking
14. Pilot tests
15. Detailed optimization methods
16. Tested parameter values during optimization
17. COVID mask effect on metabolics

Supplemental Videos

1. Videos of walking

## 1. User training

**Participants 1 and 2:**
These two users designed, built and tested the device. Testing included tuning the control gains which resulted in months of walking with a wide variety of torque profiles. Participants 1 and 2 empirically found the maximum and minimum parameter values. Participant 1 completed a nine-parameter pilot optimization which took 10 days and over 40 hours of experiment time (note 14).

**Participant 3:**
**Previous experience with exoskeletons**
Participant 3 walked in bilateral ankle-only exoskeletons in a previous optimization study. This took 5 days and roughly 20 hours of experiment time.

**Hip-knee-ankle exoskeleton fitting**
We fit the device to participant 3 on their first day. This included choosing and adjusting boots, adjusting the shank and thigh lengths to match the knee and hip joint centers, and adjusting the thigh and hip widths to comfortably and snugly fit participant 3. Once the device was sized, participant 3 walked without assistance for about 20 minutes to get used to walking with the device. Users need to adapt to the mass and impedance of the device when they first wear it.

**Training for ankle-only optimization**
After the fitting day and before the first day of optimization, this participant had an ankle-only training day. They walked in the device without assistance for 20 minutes in total. Then they walked with ankle-only torques up to 0.8 Nm/kg for a total of 20 minutes. Participant 3 then completed the ankle-only optimization which was 4 experiments on separate days for a total of 12 hours

**Training for hip-only optimization**
After the ankle-only optimization, participant 3 completed a day of hip-only assistance training. This was similar to the ankle-only training, with 20 minutes of walking with hip-only assistance up to 0.34 Nm/kg. The profile applied during training was the first condition of the first generation for optimization. Participant 3 then completed the hip-only optimization which was 4 experiments on separate days for a total of 12 hours.

Participant 3 did not train for knee-only optimization. This was done in the interest of participant time and because the participant had a relatively easy time walking with knee-only torque.

**Training for whole-leg optimization**
Participant 3 completed a training day before beginning the whole-leg optimization. We built up the assistance of the first condition of the first generation for over 20 minutes. The participant walked with assistance at 50% of the total magnitude, then increased to 75% when they were comfortable. Finally they walked with 100% once they were comfortable with 75% of the total magnitude. Participant 3 then completed 20 minutes of walking with the seed of the first generation. We measured the metabolic cost during that time. After that, we measured their metabolic cost of walking without assistance and their quiet standing metabolic cost.

Including optimization experiments, training days and the fitting day, participant 3 had 45 hours of hip-knee-ankle exoskeleton experience before beginning the whole-leg optimization experiment.

**Participant 4:**

**Previous experience with exoskeletons**

Participant 4 walked in bilateral ankle-only exoskeletons in a previous optimization study. This took 10 days and roughly 40 hours of experiment time.

**Hip-knee-ankle exoskeleton fitting**

The device was fit to participant 4 using the same protocol used for participant 3. Once the device was sized, participant 4 walked without assistance for 30 minutes to get used to the device.

**Training for two- and three-joint assistance optimization**

Participant 4 completed two training sessions before beginning the whole-leg optimization. The first session involved walking in single-joint generic assistance (the average of participants 1, 2, and 3's optimized parameters) for 30 minutes at each joint. Each of the three 30 minute segments was broken into three 10-minute segments to allow for torque ramp-up according to the following: 10 minutes at 50% torque, 10 minutes at 75% torque, 10 minutes at 100% torque. The second session focused on acclimating the subject to multi-joint assistance. The subject walked for 30 minutes in generic hip-knee-ankle assistance at 50% torque, then 30 minutes at 75% torque, and lastly 30 minutes at 100% torque.

Including optimization experiments, training days, and fitting days, participant 4 had 50 hours of exoskeleton experience before beginning the whole-leg optimization experiment.

## 2. Metabolics - single-joint and whole-leg

**Metabolic cost of each condition in W/kg. Cost shown for walking conditions is measured cost (without quiet standing subtracted). Percent reductions were calculated using the cost of walking for each condition, which is the measured cost minus the cost of quiet standing. Standard deviation shown for repeated conditions.**

| | Participant Info | Quiet Standing | No exo. | No torque | Hip-only | Knee-only | Ankle-only | Whole-leg |
|---|---|---|---|---|---|---|---|---|
| P1 | 90 kg, 187 cm, M | 1.43 +/- 0.09 | 4.09 +/- 0.15 | 4.66 +/- 0.14 | 3.64 | 4.27 | 3.71 | 2.92 |
| P2 | 60.75 kg, 170 cm, F | 1.70 +/- 0.06 | 4.93 +/- 0.26 | 6.04 +/- 0.19 | 5.12 | 5.90 | 4.61 | 3.79 |
| P3 | 81.5 kg, 176 cm, M | 1.51 +/- 0.04 | 4.36 +/- 0.19 | 5.06 +/- 0.18 | 4.10 | 4.60 | 4.08 | 3.34 |
| P4 | 62 kg, 171 cm, M | 1.43 | 4.12 | 6.01 | | | | 3.65 |

### 3. Metabolics - two-joint

**Metabolic cost of each condition in W/kg. Cost shown for walking conditions is measured cost (without quiet standing subtracted). Percent reductions were calculated using the cost of walking for each condition, which is the measured cost minus the cost of quiet standing. Standard deviation shown for repeated conditions.**

| | Participant Info | Quiet Standing | No exo. | No torque | Hip-knee | Knee-ankle | Hip-ankle |
|---|---|---|---|---|---|---|---|
| P1 | 90 kg, 187 cm, M | 0.84 +/- 0.07 | 2.71 +/- 0.02 | 3.30 +/- 0.16 | 2.31 | 2.51 | 2.35 |
| P3 | 85 kg, 176 cm, M | 1.33 +/- 0.29 | 4.02 +/- 0.61 | 4.93 +/- 0.58 | 3.90 | 3.05 | 3.61* |
| P4 | 62 kg, 171 cm, M | 1.32 +/- 0.11 | 3.76 +/- 0.30 | 4.99 +/- 0.88 | 2.95 | 4.01 | 4.04 |

During all of these tests except for P3 hip-ankle (starred), these participants were wearing a cloth mask underneath the metabolics mask due to COVID-19 safety protocols. This mask seems to have affected the measurements by causing an underestimation of metabolic cost. While these absolute changes are not ideal, we anticipate that they should be consistent across conditions, and therefore not greatly affect our estimates of percent reductions in metabolic cost from two-joint assistance. More description of the cloth mask's effect on metabolic measurements is available below in note 17. Note that for P1, hip-ankle assistance produced a larger percent reduction than hip-knee assistance because of variation in the control conditions across days (i.e. the cost of walking was generally higher for the participant on the day of hip-ankle validation compared to the day of hip-knee validation.)

## 4. Applied torques for each participant

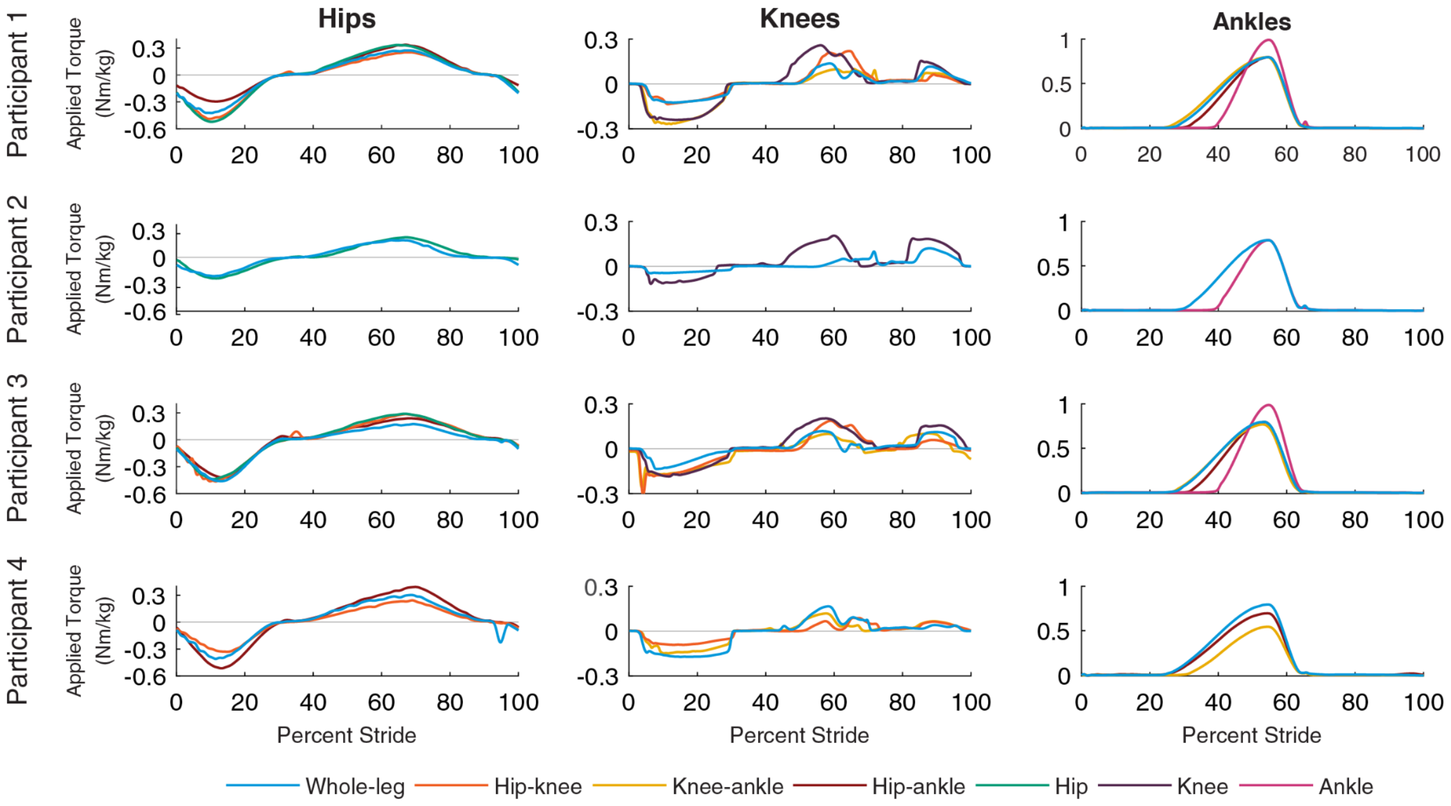


**Applied torques for each participant.** Optimized single-joint, two-joint and whole-leg exoskeleton assistance torques at the hips (left), knees (center), and ankles (right). Torques are normalized by body mass. Participant 2 and Participant 4 were unable to participate in two-joint and single-joint assistance, respectively. For the ankles, maximum torque had to be constrained to find comfortable profiles for walking. Ankle torques were limited to 1 Nm/kg for single-joint assistance, and 0.8 Nm/kg for two-joint and whole-leg assistance.

## 5. Optimized parameters and ranges - hips

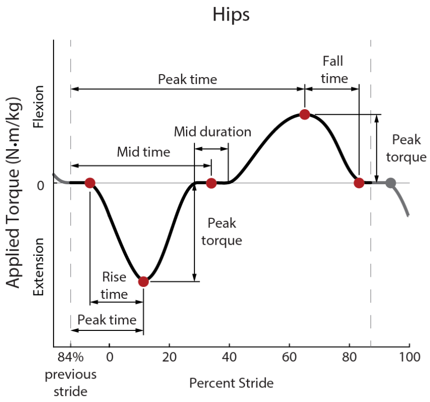


Hip parameter ranges (minimums and maximums) and initial values for hip-only assistance for P1. Whole-leg initial values based on single-joint optimized values. P2 and P3 initial values were based on optimized parameters from P1, and P4's initial values were the average of P1, P2, and P3. Times are reported as percentages of stride, with 1 being 100% of stride. Torques are reported in Nm/kg. Percent stride values are based off of hip stride timer (to have in terms of heel strike, subtract 16% from the reported time). The hip profile starts 84% of stride after heel strike because hip extension torque is active during heel strike. Resetting the hips' stride time at heel strike caused discrete jumps in hip extension torque during pilot testing.

### Hip parameter ranges (minimums and maximums) and initial values for P1.

| Hips | Hip ext. rise time | Hipe ext. peak time | Hip ext. peak torque (Nm/kg) | Mid time | Mid duration | Hip flex. Peak time | Hip flex. peak torque (Nm/kg) | Hip flex. fall time |
|---|---|---|---|---|---|---|---|---|
| Min | 0.05 | 0.15 | 0 | 0.4 | 0 | 0.65 | 0 | 0.05 |
| Initial (P1, hip-only) | 0.15 | 0.26 | 0.35 | 0.5 | 0.1 | 0.74 | 0.35 | 0.15 |
| Max | 0.3 | 0.35 | 0.7 | 0.6 | 0.3 | 0.85 | 0.6 | 0.4 |

### Optimized hip parameters for each participant and condition.

| Hips-Only | HE RT | HE PS Peak | HE Torque | mid PS | mid Dur | HF PS | HF T | HF FT |
|---|---|---|---|---|---|---|---|---|
| P1 | 0.176 | 0.259 | 0.525 | 0.472 | 0.020 | 0.814 | 0.339 | 0.244 |
| P2 | 0.167 | 0.265 | 0.237 | 0.503 | 0.017 | 0.824 | 0.230 | 0.228 |
| P3 | 0.147 | 0.263 | 0.449 | 0.482 | 0.011 | 0.820 | 0.290 | 0.270 |
| Average | 0.164 | 0.262 | 0.404 | 0.486 | 0.016 | 0.819 | 0.286 | 0.247 |
| | | | | | | | | |
| Hip-knee | HE RT | HE PS Peak | HE Torque | mid PS | mid Dur | HF PS | HF T | HF FT |
| P1 | 0.202 | 0.261 | 0.490 | 0.471 | 0.013 | 0.837 | 0.252 | 0.235 |
| P3 | 0.190 | 0.264 | 0.457 | 0.487 | 0.033 | 0.827 | 0.283 | 0.279 |
| P4 | 0.204 | 0.299 | 0.485 | 0.470 | 0.011 | 0.831 | 0.341 | 0.227 |
| Average | 0.199 | 0.275 | 0.477 | 0.476 | 0.019 | 0.832 | 0.292 | 0.247 |
| | | | | | | | | |
| Hip-ankle | HE RT | HE PS Peak | HE Torque | mid PS | mid Dur | HF PS | HF T | HF FT |
| P1 | 0.206 | 0.274 | 0.301 | 0.488 | 0.034 | 0.826 | 0.338 | 0.237 |
| P3 | 0.183 | 0.291 | 0.434 | 0.457 | 0.000 | 0.846 | 0.222 | 0.237 |
| P4 | 0.174 | 0.287 | 0.510 | 0.468 | 0.004 | 0.847 | 0.383 | 0.220 |
| Average | 0.188 | 0.284 | 0.415 | 0.471 | 0.013 | 0.839 | 0.314 | 0.231 |
| | | | | | | | | |
| Whole-leg | HE RT | HE PS Peak | HE Torque | mid PS | mid Dur | HF PS | HF T | HF FT |
| P1 | 0.196 | 0.255 | 0.425 | 0.478 | 0.025 | 0.824 | 0.283 | 0.228 |
| P2 | 0.187 | 0.260 | 0.211 | 0.473 | 0.014 | 0.800 | 0.207 | 0.203 |
| P3 | 0.180 | 0.281 | 0.474 | 0.463 | 0.000 | 0.841 | 0.173 | 0.210 |
| P4 | 0.181 | 0.282 | 0.407 | 0.463 | 0.014 | 0.822 | 0.302 | 0.243 |
| Average | 0.186 | 0.270 | 0.379 | 0.469 | 0.013 | 0.822 | 0.241 | 0.221 |

## Optimized parameters and ranges - knees

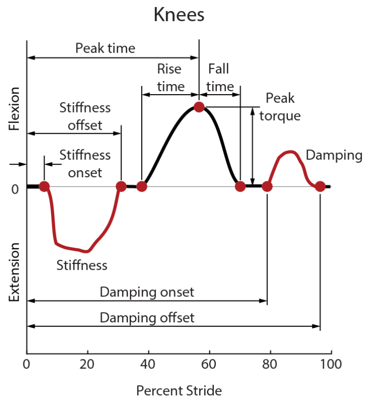


Knee parameter ranges (minimums and maximums) and initial values for knee-only assistance for P1. Whole-leg initial values based on single-joint optimized values. P2 and P3 initial values were based on optimized parameters from P1, and P4's initial values were the average of P1, P2, and P3. Times are reported as percentages of stride, with 1 being 100% of stride. Torques are reported in Nm/kg. Stiffness is in units of (Nm/kg)/deg, damping coefficient in units of (Nm/kg)/(deg/sec).
*Knee flexion torque tracking was worse than other directions of actuation. These parameters define the desired torque, but measured torque plots give a better estimate of the effective torque that was applied to the user.

### Knee parameter ranges (minimums and maximums) and initial values for P1, knee only

| Knees | Stiffness onset | Stiffness *k* | Stiffness offset | Knee flex. rise time | Knee flex. peak time | Knee flex. peak torque | Knee flex. fall time | Damping onset | Damping coefficient *b* | Damping offset |
|---|---|---|---|---|---|---|---|---|---|---|
| Min | 0.001 | 0 | 0.1 | 0.1 | 0.35 | 0 | 0.05 | 0.65 | 0 | 0.7 |
| Initial | 0.03 | 0.0198 | 0.25 | 0.25 | 0.55 | 0.196 | 0.08 | 0.75 | 2 | 0.95 |
| Max | 0.2 | 0.04 | 0.4 | 0.35 | 0.65 | 0.4 | 0.25 | 0.9 | 4 | 0.999 |

### Optimized knee parameters for each participant and condition.

| Knees-Only | KE k on | KE k | KE k Off | KF RT | KF P time | KF Torque* | KF FT | Damp on | Damp *b* | Damp Off |
|---|---|---|---|---|---|---|---|---|---|---|
| P1 | 0.025 | 0.014 | 0.271 | 0.205 | 0.588 | 0.329 | 0.094 | 0.812 | 2.351 | 0.968 |
| P2 | 0.029 | 0.006 | 0.247 | 0.219 | 0.591 | 0.258 | 0.075 | 0.766 | 2.071 | 0.964 |
| P3 | 0.022 | 0.007 | 0.284 | 0.218 | 0.592 | 0.250 | 0.117 | 0.818 | 2.107 | 0.977 |
| Average | 0.026 | 0.009 | 0.267 | 0.214 | 0.590 | 0.279 | 0.095 | 0.798 | 2.176 | 0.969 |
| | | | | | | | | | | |
| Hip-Knee | KE k on | KE k | KE k Off | KF RT | KF P time | KF Torque* | KF FT | Damp on | Damp *b* | Damp Off |
| P1 | 0.029 | 0.016 | 0.288 | 0.171 | 0.620 | 0.258 | 0.088 | 0.810 | 0.910 | 0.974 |
| P3 | 0.032 | 0.010 | 0.284 | 0.150 | 0.605 | 0.212 | 0.102 | 0.789 | 0.904 | 0.990 |
| P4 | 0.023 | 0.008 | 0.297 | 0.157 | 0.625 | 0.227 | 0.093 | 0.782 | 0.827 | 0.985 |
| Average | 0.028 | 0.011 | 0.290 | 0.159 | 0.617 | 0.233 | 0.094 | 0.794 | 0.881 | 0.983 |
| | | | | | | | | | | |
| Knee-Ankle | KE k on | KE k | KE k Off | KF RT | KF P time | KF Torque* | KF FT | Damp on | Damp *b* | Damp Off |
| P1 | 0.027 | 0.019 | 0.276 | 0.174 | 0.621 | 0.116 | 0.093 | 0.807 | 1.270 | 0.998 |
| P3 | 0.029 | 0.007 | 0.300 | 0.167 | 0.604 | 0.121 | 0.073 | 0.787 | 2.029 | 0.938 |
| P4 | 0.021 | 0.012 | 0.289 | 0.183 | 0.581 | 0.197 | 0.085 | 0.783 | 1.091 | 0.968 |
| Average | 0.026 | 0.013 | 0.288 | 0.175 | 0.602 | 0.145 | 0.084 | 0.792 | 1.463 | 0.968 |
| | | | | | | | | | | |
| Whole-leg | KE k on | KE k | KE k Off | KF RT | KF P time | KF Torque* | KF FT | Damp on | Damp *b* | Damp Off |
| P1 | 0.027 | 0.016 | 0.283 | 0.165 | 0.611 | 0.203 | 0.093 | 0.807 | 1.348 | 0.985 |
| P2 | 0.028 | 0.008 | 0.290 | 0.155 | 0.609 | 0.122 | 0.104 | 0.794 | 0.973 | 0.964 |
| P3 | 0.025 | 0.010 | 0.293 | 0.172 | 0.588 | 0.175 | 0.085 | 0.801 | 1.340 | 0.951 |
| P4 | 0.019 | 0.013 | 0.288 | 0.152 | 0.593 | 0.198 | 0.095 | 0.797 | 0.495 | 0.961 |
| Average | 0.024 | 0.012 | 0.289 | 0.161 | 0.600 | 0.174 | 0.094 | 0.800 | 1.039 | 0.965 |

## Optimized parameters and ranges - ankles

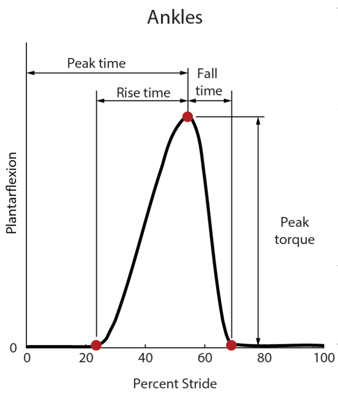


Ankle parameter ranges (minimums and maximums) and initial values for ankle-only assistance for P1. Whole-leg initial values based on single-joint optimized values. P2 and P3 initial values were based on optimized parameters from P1, and P4's initial values were the average of P1, P2, and P3. Times are reported as percentages of stride, with 1 being 100% of stride. Torques are reported in Nm/kg. The maximum allowed peak torque was larger for single-joint than multi-joint conditions, because some users found the magnitude to be uncomfortable and destabilizing, which was worse during multi-joint assistance.
*Ankle rise time was reinitialized as 0.3 for whole-leg assistance, because users found the optimized rise time from ankle-only assistance to be short and uncomfortable.

### Ankle parameter ranges (minimums and maximums) and initial values for P1

| Ankles | Peak torque | Peak time | Rise time | Fall time* |
|---|---|---|---|---|
| Min | 0 | 0.35 | 0.1 | 0.05 |
| Initial | 0.5 | 0.48 | 0.3 | 0.1 |
| Max | 1 (single-joint), 0.8 (whole-leg) | 0.55 | 0.4 | 0.2 |

*Torque was limited to be applied no later than 65% of stride, so, for example, if peak time was at its latest allowed value (55% of stride), fall time was limited to be 10% of stride.

### Optimized ankle parameters for each participant and condition.

| Ankles-Only | Peak torque | Peak time | Rise time | Fall time* |
|---|---|---|---|---|
| P1 | 1.000 | 0.550 | 0.177 | 0.184 |
| P2 | 0.804 | 0.550 | 0.182 | 0.200 |
| P3 | 1.000 | 0.550 | 0.175 | 0.200 |
| Average | 0.935 | 0.550 | 0.178 | 0.195 |
| | | | | |
| KA Ankles | Peak torque | Peak time | Rise time | Fall time* |
| P1 | 0.800 | 0.546 | 0.320 | 0.200 |
| P3 | 0.800 | 0.547 | 0.304 | 0.182 |
| P4 | 0.800 | 0.550 | 0.269 | 0.100 |
| Average | 0.800 | 0.548 | 0.297 | 0.161 |
| | | | | |
| HA Ankles | Peak torque | Peak time | Rise time | Fall time* |
| P1 | 0.800 | 0.550 | 0.266 | 0.194 |
| P3 | 0.800 | 0.538 | 0.246 | 0.181 |
| P4 | 0.693 | 0.550 | 0.324 | 0.122 |
| Average | 0.764 | 0.546 | 0.278 | 0.165 |
| | | | | |
| HKA Ankles | Peak torque | Peak time | Rise time | Fall time* |
| P1 | 0.800 | 0.550 | 0.306 | 0.190 |
| P2 | 0.800 | 0.550 | 0.282 | 0.173 |
| P3 | 0.800 | 0.538 | 0.292 | 0.189 |
| P4 | 0.800 | 0.550 | 0.328 | 0.111 |
| Average | 0.800 | 0.547 | 0.302 | 0.166 |

## 6. Applied exoskeleton power

**Net Power (W/kg)**

| | Single Joint | | | Whole leg | | | | Hip-Ankle | | Hip-Knee | | Knee-Ankle | |
|---|---|---|---|---|---|---|---|---|---|---|---|---|---|
| | Hip only | Knee only | Ankle Only | Hip | Knee | Ankle | Total | Hip | Ankle | Hip | Knee | Knee | Ankle |
| P1 | 0.550 | 0.347 | 0.753 | 0.529 | 0.076 | 0.204 | 0.809 | 0.529 | 0.252 | 0.555 | 0.278 | 0.107 | 0.325 |
| P2 | 0.401 | 0.127 | 0.598 | 0.362 | -0.107 | 0.495 | 0.750 | | | | | | |
| P3 | 0.578 | 0.177 | 0.572 | 0.510 | 0.032 | -0.035 | 0.507 | 0.586 | 0.121 | 0.730 | 0.312 | 0.056 | 0.237 |
| P4 | | | | 0.650 | 0.188 | 0.159 | 0.998 | 0.816 | 0.291 | 0.767 | 0.018 | 0.187 | 0.501 |
| Avg | **0.510** | **0.217** | **0.641** | **0.513** | **0.047** | **0.206** | **0.766** | **0.644** | **0.221** | **0.684** | **0.203** | **0.117** | **0.354** |

**Positive Power (W/kg)**

| | Single Joint | | | Whole leg | | | | Hip-Ankle | | Hip-Knee | | Knee-Ankle | |
|---|---|---|---|---|---|---|---|---|---|---|---|---|---|
| | Hip only | Knee only | Ankle Only | Hip | Knee | Ankle | Total | Hip | Ankle | Hip | Knee | Knee | Ankle |
| P1 | 0.571 | 0.489 | 0.768 | 0.541 | 0.206 | 0.298 | 1.044 | 0.558 | 0.323 | 0.573 | 0.354 | 0.207 | 0.402 |
| P2 | 0.404 | 0.355 | 0.642 | 0.371 | 0.048 | 0.652 | 1.071 | | | | | | |
| P3 | 0.599 | 0.399 | 0.637 | 0.528 | 0.185 | 0.253 | 0.966 | 0.620 | 0.335 | 0.750 | 0.388 | 0.189 | 0.358 |
| P4 | | | | 0.705 | 0.279 | 0.319 | 1.302 | 0.871 | 0.391 | 0.801 | 0.184 | 0.349 | 0.545 |
| Avg | **0.525** | **0.414** | **0.682** | **0.536** | **0.179** | **0.380** | **1.096** | **0.683** | **0.350** | **0.708** | **0.309** | **0.248** | **0.435** |

**Negative Power (W/kg)**

| | Single Joint | | | Whole leg | | | | Hip-Ankle | | Hip-Knee | | Knee-Ankle | |
|---|---|---|---|---|---|---|---|---|---|---|---|---|---|
| | Hip only | Knee only | Ankle Only | Hip | Knee | Ankle | Total | Hip | Ankle | Hip | Knee | Knee | Ankle |
| P1 | -0.021 | -0.142 | -0.014 | -0.029 | -0.130 | -0.107 | -0.265 | -0.029 | -0.071 | -0.017 | -0.075 | -0.097 | -0.076 |
| P2 | -0.006 | -0.228 | -0.044 | -0.009 | -0.155 | -0.156 | -0.321 | | | | | | |
| P3 | -0.021 | -0.222 | -0.065 | -0.018 | -0.153 | -0.288 | -0.459 | -0.034 | -0.214 | -0.021 | -0.077 | -0.133 | -0.120 |
| P4 | | | | -0.054 | -0.090 | -0.160 | -0.304 | -0.055 | -0.100 | -0.034 | -0.167 | -0.161 | -0.044 |
| Avg | **-0.016** | **-0.198** | **-0.041** | **-0.027** | **-0.132** | **-0.178** | **-0.337** | **-0.029** | **-0.071** | **-0.024** | **-0.106** | **-0.131** | **-0.080** |

**Calculation**

We calculated the joint power by multiplying the applied torque by the joint angular velocity, and we calculated the angular velocity as the time derivative of the joint angle low pass filtered at 50 Hz. We averaged the power over the last 5 minutes to determine the net power, positive power and negative power. The reported values are the total power applied to both legs.

**Discussion**

Whole leg assistance resulted in less applied torque per joint than single-joint assistance. Knee and ankle assistance applied considerably less power during whole leg assistance than single-joint assistance, but hip assistance applied a similar amount of power for single-joint, two-joint and whole-leg assistance. Participants may be able to accept more joint power during single joint assistance because they can offload it to assist other joints. This may be seen in kinematic adaptations and muscle activity reductions at non-assisted joints The net applied power mimics the trend of metabolic reductions, where there are larger metabolic reductions with more exoskeleton power. However, the net power would suggest a smaller metabolic reduction for whole leg assistance than what we saw. The positive power also follows that trend, but the positive knee power would suggest a larger metabolic reduction than what we saw.

## 7. Kinematics for each participant

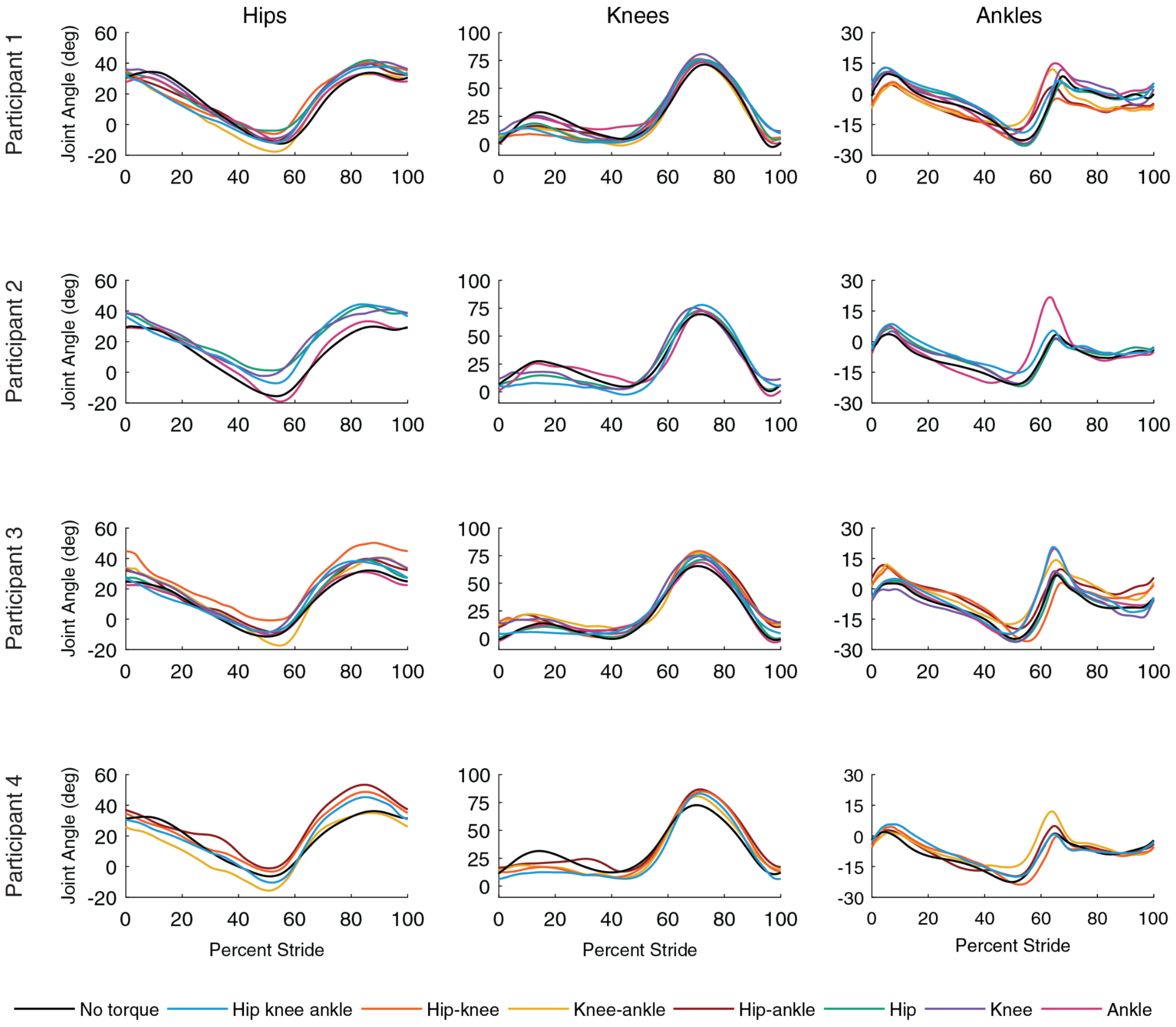


**Average joint kinematics for each participant**. Average joint angle as a percentage of stride at the hips (left), knees (center), and ankles (right) for each condition (denoted by color) for each participant. All single-joint and whole-leg conditions for Participants 1, 2, and 3 were tested on the same day to reduce changes in alignment between user and device. Two-joint and Participant 4 three-joint conditions were each collected individually. Positive joint angles are in flexion for the hips, in flexion for the knees, and in plantarflexion for the ankles.

## 8. Stride frequency for each participant

**Stride frequency (Hz) for each condition for each participant.**

| Participant | No exo. | No torque | Hip-only | Knee-only | Ankle-only | Whole-leg | Hip-knee | Knee-ankle | Hip-ankle |
|---|---|---|---|---|---|---|---|---|---|
| P1 | 0.827+/-0.013 | 0.816+/-0.010 | 0.857 | 0.870 | 0.758 | 0.828 | 0.855 | 0.797 | 0.861 |
| P2 | 0.981+/-0.020 | 0.968+/-0.016 | 0.961 | 0.987 | 0.960 | 0.975 | | | |
| P3 | 0.838+/-0.103 | 0.840+/-0.022 | 0.913 | 0.869 | 0.872 | 0.944 | 0.934 | 0.835 | 0.961 |
| P4 | 0.991+/-0.025 | 1.014+/-0.023 | | | | 0.963 | 1.055 | 1.065 | 1.071 |
| Average | **0.909+/-0.040** | **0.910+/-0.018** | **0.910** | **0.909** | **0.863** | **0.927** | **0.948** | **0.899** | **0.964** |

Stride frequency was calculated using heel strikes detected from ground reaction forces. At the group level, there were no significant changes in stride frequency between conditions, but large variability between participants was present. Stride frequency seemed to indicate maladaptations to assistance at times. For example, P3 first adapted to whole-leg assistance with a walking strategy that had a 25% increase in stride frequency, which resulted in a metabolic reduction that was worse than expected based on previous participants. After being instructed to take longer steps, the participant converged on a walking strategy that had a stride frequency of 0.944 Hz, which was a 12.33% increase compared to the no torque condition, but was lower than the first maladaptation and corresponded to a better metabolic reduction.

## 9. Muscle activity - participant 1

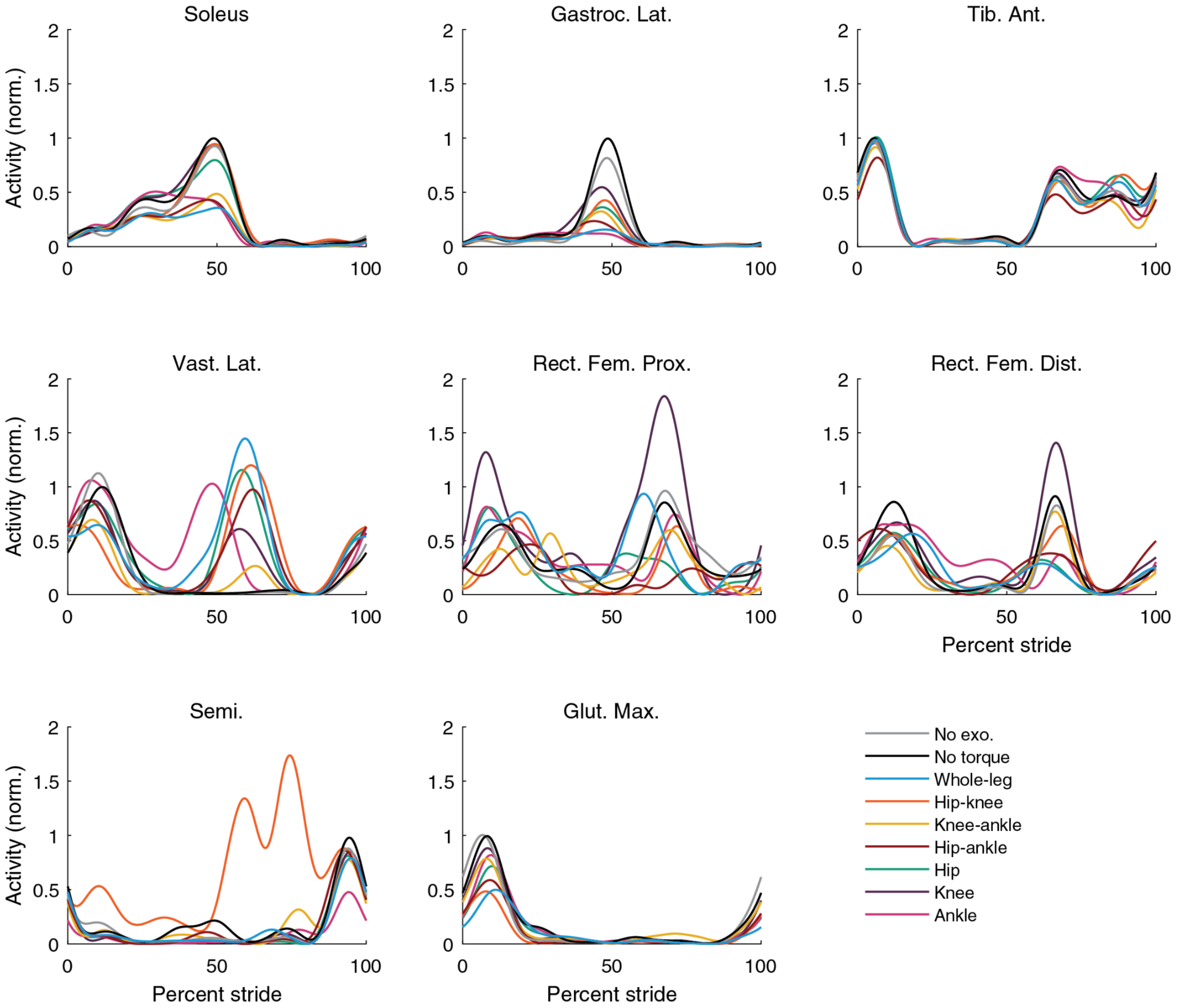


**Muscle activity for participant 1.** Muscle activity measured during walking using surface EMG for each condition. The EMG signal was filtered, averaged, had baseline activity removed to eliminate noise, and normalized to the peak value of walking in the exoskeleton without assistance (black). During hip-knee assistance (green-yellow dashed), it seems that the EMG sensor for the semitendinosus (bottom-left) was dislodged, leading to very noisy measurements.

## Muscle activity - participant 2

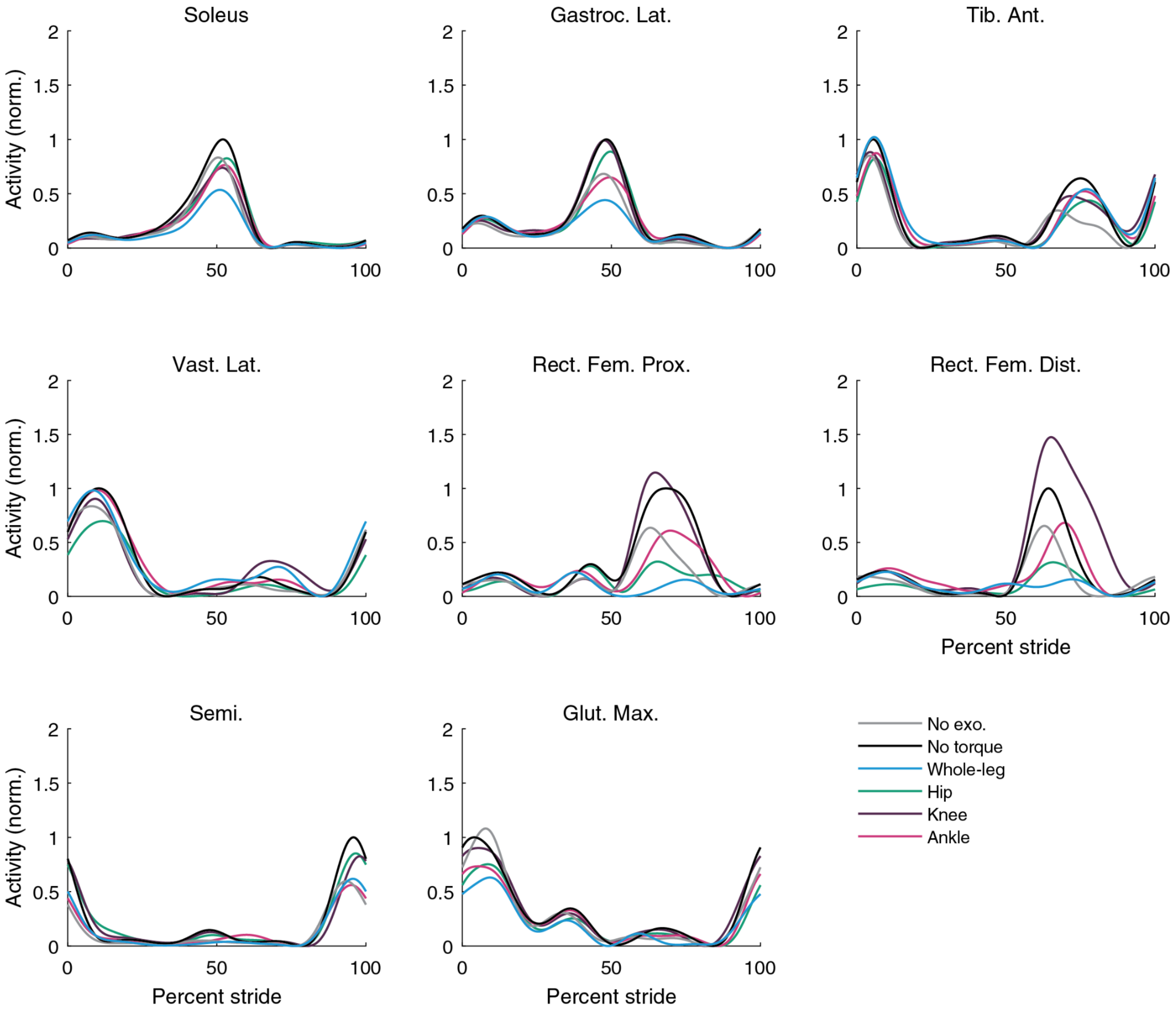


**Muscle activity for participant 2.** Muscle activity measured during walking using surface EMG for each condition. The EMG signal was filtered, averaged, had baseline activity removed to eliminate noise, and normalized to the peak value of walking in the exoskeleton without assistance (black).

## Muscle activity - participant 3

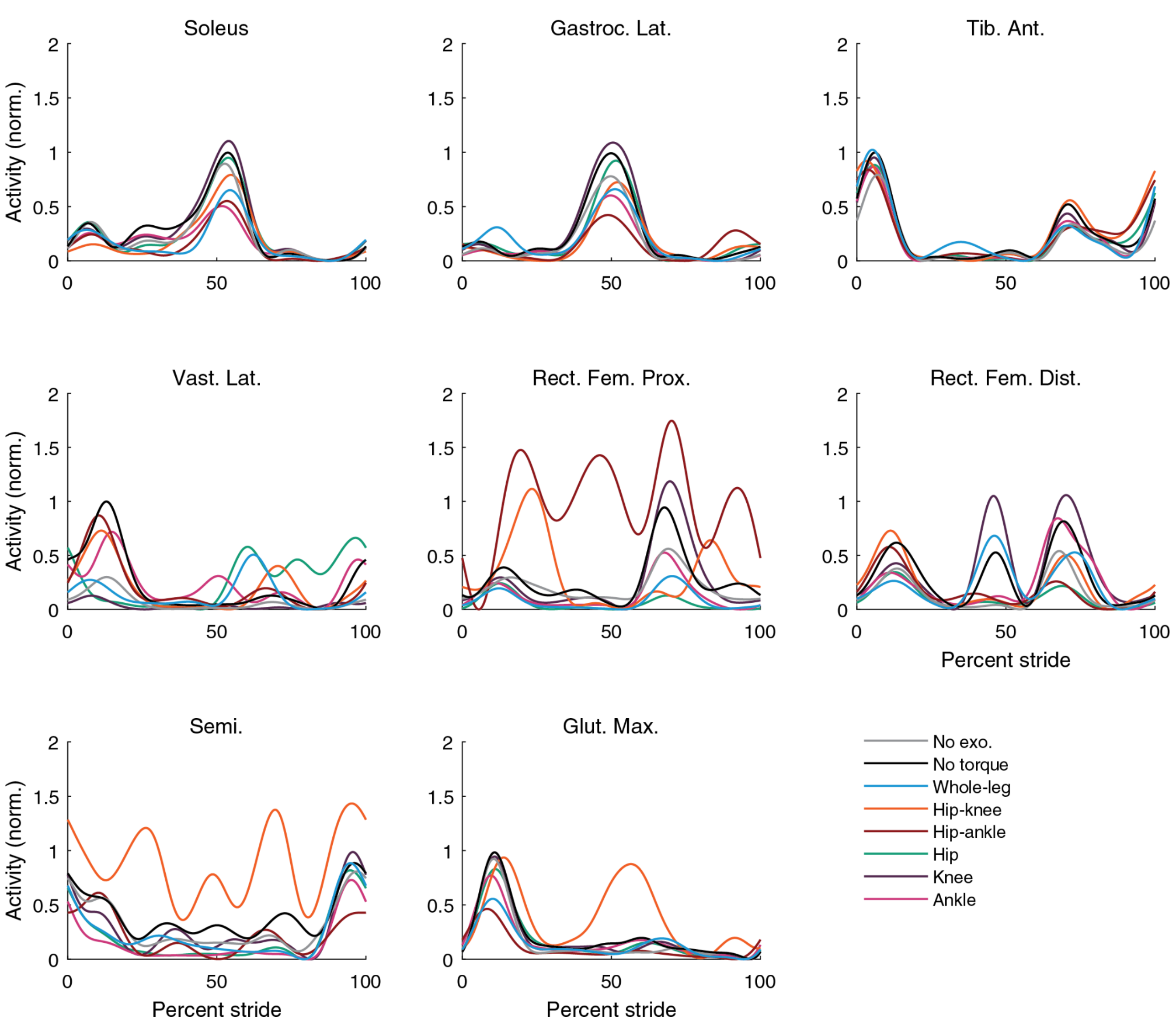


**Muscle activity for participant 3.** Muscle activity measured during walking using surface EMG for each condition. The EMG signal was filtered, averaged, had baseline activity removed to eliminate noise, and normalized to the peak value of walking in the exoskeleton without assistance (black). During hip-ankle and hip-knee assistance, it seems that the rectus femoris proximal and semitendinosus sensors became dislodged, respectively. This subject does not have EMG reported for their knee-ankle assistance due to a technical difficulty during data collection in that individual experiment.

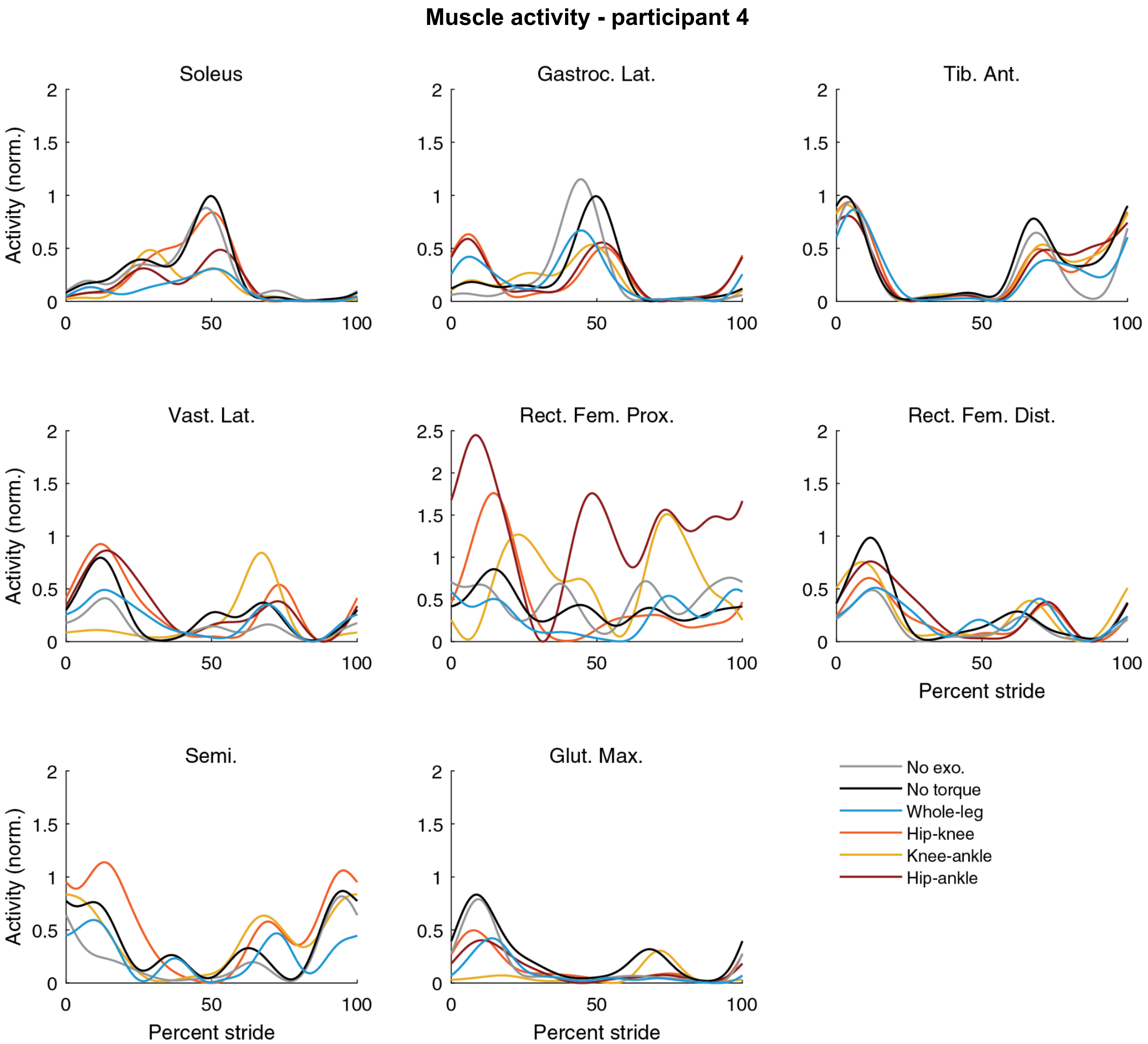


**Muscle activity for participant 4**. Muscle activity measured during walking using surface EMG for each condition. The EMG signal was filtered, averaged, had baseline activity removed to eliminate noise, and normalized to the peak value of walking in the exoskeleton without assistance (black). During hip-ankle assistance, it seems that the rectus femoris proximal sensor became dislodged, leading to noisy signals.

### 10. Mobile device mass estimate

| | | | | | |
|---|---|---|---|---|---|
| | Hip | Knee | Ankle | | |
| Peak torque of average optimized whole-leg assistance (Nm/kg) | 0.36 | 0.11 | 0.79 | | |
| | | | | | |
| | Hip | Knee | Ankle | | User mass (kg) |
| Peak torque requirements (Nm) | 32 | 10 | 71 | | 90 |
| | | | | | |
| Hip Exoskeletons | Average | Bleex* (44) | Sant'Anna (45) | Samsung (11) | Panasonic (46) |
| Peak Torque (Nm) | | 150 | 35 | 11 | 20 |
| Total Mass (kg) | | 41 | 4.2 | 2.8 | 9.3 |
| Mass for bilateral hips | | 20.5 | 4.2 | 2.8 | 9.3 |
| Torque Density (Nm/kg) | 5 | 7 | 8 | 4 | 2 |
| | | | | | |
| Knee Exoskeletons | Average | Bleex* (44) | Northwestern (43) | RoboKnee (47) | |
| Peak Torque (Nm) | | 125 | 80 | 133 | |
| Total Mass (kg) | | 41 | 4.1 | 3 | |
| Mass for bilateral knees (kg) | | 13.7 | 8.2 | 6 | |
| Torque Density (Nm/kg) | 13.7 | 9.1 | 9.8 | 22.2 | |
| | | | | | |
| Ankle Exoskeletons | Average | Bleex* (44) | MIT** (9) | Achilles (48) | |
| Peak Torque | | 175 | 120 | 68 | |
| Total Mass (kg) | | 41 | 4 | 8.2 | |
| Mass for bilateral ankles (kg) | | 6.8 | 4 | 8.2 | |
| Torque Density (Nm/kg) | 21.3 | 25.6 | 30.0 | 8.3 | |
| | | | | | |
| Estimated mobile device mass using average torque densities | | Estimates were taken for mobile devices from device comparison in Bryan et al. 2020 (28).<br>*For BLEEX, assumed device mass distribution of 1/2 of weight at the hips, 1/3 at the knees, 1/6 at the ankles.<br>**For MIT ankle, torque density is calculated using total system mass, not just joint mass as we did in Bryan et al. 2020 | | | |
| Device Mass: hips | 5.94 | | | | |
| Device mass: knees | 0.72 | | | | |
| Device mass: ankles | 3.34 | | | | |
| **Total device mass estimate (kg)** | **10.00** | | | | |

We estimated what a mobile hip-knee-ankle exoskeleton capable of our optimized whole-leg assistance torques might weigh. This estimate is intended to understand how well the findings from our study with our exoskeleton emulator (worn mass: 13.5 kg) could translate to a mobile device. To do this, we calculated an average torque density for each joint using the published torque capabilities and device masses from mobile exoskeletons. We then divided the peak torque of each joint of our optimized whole-leg profile for a 90 kg user by these average torque densities to estimate the mass of a mobile device capable of applying these torques at each joint.

## 11. Experiment log - generations and reductions per day

| P1 Hip-Only | | |
|---|---|---|
| **Day** | **Generations** | **Reduction** |
| 1 | 1-2 | Not Tested |
| 2 | 3-5 | 25% |
| 3 | 6-8 | 22% |
| 4 | 9-11 | 22% |
| 5 | 12-14 | 29% |
| 6 | 15-16 | 28% |
| 7 | 17-19 | 30% |
| 8 | 20-22 | 32% |
| 9 | 23-25 | 25% |
| 10 | Validation | 30% |

| P2 Hip-Only | | |
|---|---|---|
| **Day** | **Generations** | **Reduction** |
| 1 | 1-3 | 19% |
| 2 | 4-6 | 17% |
| 3 | 7-9 | 23% |
| 4 | Validation | 24% |

| P3 Hip-Only | | |
|---|---|---|
| **Day** | **Generations** | **Reduction** |
| 1 | 1-3 | 20% |
| 2 | 4-6 | 31% |
| 3 | 7-9 | 28% |
| 4 | Validation | 24% |

For hip-only, each generation was 20 minutes of walking.

| P1 Knee-Only | | |
|---|---|---|
| **Day** | **Gens** | **Reduction** |
| 1 | 1-3 | 12% |
| 2 | 4-6 | 14% |
| 3 | 7-9 | 13% |
| 4 | 10-12 | 11% |
| 5 | Validation | 18% |

| P2 Knee-Only | | |
|---|---|---|
| **Day** | **Gens** | **Reduction** |
| 1 | 1-3 | 7% |
| 2 | 4-6 | Not Tested |
| 3 | 7-9 | 8% |
| 4 | Validation | 5% |

| P3 Knee-Only | | |
|---|---|---|
| **Day** | **Gens** | **Reduction** |
| 1 | 1-3 | Not Tested |
| 2 | 4-6 | -2% |
| 3 | 7-9 | 11% |
| 4 | Validation | 16% |

For knee-only, each generation was 20 minutes of walking.

| P1 Ankle-Only | | |
|---|---|---|
| **Day** | **Gens** | **Reduction** |
| 1 | 1-4 | 23% |
| 2 | 5-8 | 26% |
| 3 | 9-12 | 33% |
| 4 | Validation | 30% |

| P2 Ankle-Only | | |
|---|---|---|
| **Day** | **Gens** | **Reduction** |
| 1 | 1-4 | 27% |
| 2 | 5-8 | 30% |
| 3 | 9-12 | 38% |
| 4 | Validation | 28% |

| P3 Ankle-Only | | |
|---|---|---|
| **Day** | **Gens** | **Reduction** |
| 1 | 1-4 | 11% |
| 2 | 5-8 | 10% |
| 3 | 9-12 | 22% |
| 4 | Validation | 31% |

For ankle-only, each generation was 16 minutes of walking.

| P1 Whole-Leg | | |
|---|---|---|
| **Day** | **Gens** | **Reduction** |
| 1 | 1-3 | 38% |
| 2 | 4-5 | 44% |
| 3 | 6-8 | 42% |
| 4 | 9-11 | 40% |
| 5 | 12-13 | 43% |

| P2 Whole-Leg | | |
|---|---|---|
| **Day** | **Gens** | **Reduction** |
| 1 | 1-3 | 39% |
| 2 | 4-6 | 41% |
| 3 | 7-9 | 47% |
| 4 | Val. | 53% |

| P3 Whole-Leg | | |
|---|---|---|
| **Day** | **Gens** | **Reduction** |
| *1** | *1-3* | *32%* |
| *2** | *4-6* | *15%* |
| *3** | *7-9* | *15%* |
| 1 | 1-3 | 30% |
| 2 | 4-6 | 37% |

| P4 Whole-Leg | | |
|---|---|---|
| **Day** | **Gens** | **Reduction** |
| 1 | 1-3 | 43% |
| 2 | 4-6 | 44% |
| 3 | 7-9 | 52% |
| 4 | Val. | 51% |

| | | | | | | | | | | | | | | |
|---|---|---|---|---|---|---|---|---|---|---|---|---|---|---|
| 6 | Val. | **51%** | | | | | | 3 | 7-9 | 41% | | | | |
| | | | | | | | | 4 | Val. | 46% | | | | |

For whole-leg, each generation was 26 minutes of walking.

| **P1 Hip-Knee** | | | | **P3 Hip-Knee** | | | | **P4 Hip-Knee** | | |
|---|---|---|---|---|---|---|---|---|---|---|
| **Day** | **Gens** | **Reduction** | | **Day** | **Gens** | **Reduction** | | **Day** | **Gens** | **Reduction** |
| 1 | 1-4 | 36% | | 1 | 1-4 | 27% | | 1 | 1-4 | 28% |
| 2 | 5-8 | Not tested | | 2 | 5-8 | Not tested | | 2 | 5-8 | 34% |
| 3 | Validation | 34% | | 3 | Validation | 29% | | 3 | Validation | 37% |

| **P1 Knee-Ankle** | | | | **P3 Knee-Ankle** | | | | **P4 Knee-Ankle** | | |
|---|---|---|---|---|---|---|---|---|---|---|
| **Day** | **Gens** | **Reduction** | | **Day** | **Gens** | **Reduction** | | **Day** | **Gens** | **Reduction** |
| 1 | 1-4 | 37% | | 1 | 1-4 | 21% | | 1 | 1-4 | 46% |
| 2 | 5-8 | Not tested | | 2 | 5-8 | 26% | | 2 | 5-8 | 34% |
| 3 | Validation | 35% | | 3 | Validation | 40% | | 3 | Validation | 36% |

| **P1 Hip-Ankle** | | | | **P3 Hip-Ankle** | | | | **P4 Hip-Ankle** | | |
|---|---|---|---|---|---|---|---|---|---|---|
| **Day** | **Gens** | **Reduction** | | **Day** | **Gens** | **Reduction** | | **Day** | **Gens** | **Reduction** |
| 1 | 1-4 | Not tested | | 1 | 1-4 | Not tested | | 1 | 1-4 | 49% |
| 2 | 5-8 | Not tested | | 2 | 5-8 | Not tested | | 2 | 5-8 | 45% |
| 3 | Validation | 42% | | 3 | Validation | 49% | | 3 | Validation | 36% |

For optimization of hip-knee assistance, each generation was 24 minutes of walking. For hip-ankle and knee-ankle assistance, each generation was 22 minutes of walking.

After each optimization was finished, if time allowed, we did a *short validation* to monitor convergence. This is in contrast to a *validation*, which sought to most accurately assess optimized assistance efficacy (e.g. for main text Figure 2) and was only conducted after all generations had been completed for a given condition (protocol described in main text). For short validations, we measured  the metabolic cost over 6 minutes of walking in the means of the generation that was just spawned (e.g. on Day 1 we optimized generations 1-3, and then tested the means of the 4th generation), measured the cost of walking in the device with no torque for 6 minutes, and measured quiet standing for 6 minutes, to calculate an estimate of the metabolic reduction from assistance, with the understanding that these measurements would be noisier than our full validations. On some days, there wasn't the experimental time to complete this measurement. For the two-joint validations, we didn't take as many short validations after optimization because we were more confident in our experimental protocol, so we felt less of a need to spend time monitoring the convergence.

*For P3, we restarted the whole-leg optimization after 3 days because of low metabolic reductions and an increase in step frequency of 25% with assistance. This data is shown in italics in the table for P3. We believe they maladapted to the assistance and the optimizer may have been stuck in a local minima that enforced the increased stride frequency, possibly related to the large hip torques. When we restarted the optimization, we coached participant 3 to "walk normally" and "take longer steps".

## 12. Sample size and power analysis

*Limitations due to participant time*

This was an extremely arduous experiment with long optimization times. P1 logged 134 experiment hours as a participant, P2 logged 50 hours as a participant, P3 logged 73.5 hours as a participant, and P4 logged 51 hours as a participant all for this protocol. Between optimization and validation days, this manuscript required 91 separate experiments. This doesn't include the pilot testing from P1 and P2, and also excludes new protocols that followed the completion of this study.

*Consistency across users*

It seems that with the extensive training and optimization times we were able to demonstrate relatively consistent reductions across users. Reductions had a range of 6% for the hips, 13% for the knees, 3% for the ankles, 8% for hip-knee, 5% for knee-ankle, 13% for hip-ankle, and 7% for whole-leg assistance. Improving our sample size could improve our accuracy of the exact estimate of the reductions.

*External conditions*

The COVID-19 pandemic hit our region following the completion of the single-joint and whole-leg optimizations for P3. With the COVID restrictions it became more difficult to do experiments due to distancing protocols, work-from-home protocols, and restrictions on who was able to access the lab. Because of these circumstances, P2 was unable to complete their two-joint optimization, so we opted to recruit a fourth participant to complete three- and two-joint optimization in their place.

*Individual participant data*

We recommend looking at the participant-level data included here in the supplementary for a more detailed view of each measured outcome. Due to our sample size, some of the smaller changes in optimized torques, kinematics, and EMG are harder to discern if they are significant. These changes could be indicative of different users adopting different walking strategies, or might approach a mean strategy as more participants walk with assistance. In the future, a study with more participants could investigate the small differences in kinematics and muscle activity seen here.

*Power analysis*
Power analysis was conducted to evaluate the minimum metabolic reduction that we can confidently detect given our sample size of three participants. Because our sample was too small to assume a normal distribution, we used a standard deviation of 7.3% in metabolic reductions from Zhang et al. 2017 which was calculated over 11 participants. This is likely a conservative estimate because we have since increased our training times and optimization times to ensure participant convergence, which we expect would shrink the deviation in reductions. Our detectable change was calculated using Matlab's "sampsizepwr" tool:

```
meanRef = 0; % no reduction
sigmaRef = 7.4; % stdev of percent reduction for Zhang et al. 2017
power = 0.8; % 80% confident
N = 4; % number of samples (in this case, participants)
p1out = sampsizepwr('t',[meanRef sigmaRef],[], power, N)
```

This gives a detectable change of 15.75, meaning with a sample size of 4 participants and assuming this deviation, we have a statistical power of at least 80% for metabolic reductions of 16% and larger. Using the same calculation, we are 90% confident in reductions greater than 19%, and are 99% confident in reductions greater than 25%. For conditions testing three participants, we are 80% confident in reductions 24% and larger, 90% confident in reductions 29% and larger, and 99% confident in reductions 41% and larger.

## 13. Torque-tracking

### Torque-tracking RMS error between average desired profile and average measured profile

| RMS error of applied torque for each condition (Nm) | P1 | P2 | P3 | Average (Nm) |
|---|---|---|---|---|
| Whole-leg, hips | 0.81 | 0.58 | 0.63 | 0.7 |
| Whole-leg, knees | 3.38 | 2.28 | 2.73 | 2.8 |
| Whole-leg, ankle | 0.28 | 0.46 | 0.35 | 0.4 |
| | | | | |
| Hips-only | 0.56 | 0.89 | 0.42 | 0.6 |
| Knees- only | 4.42 | 2.58 | 2.88 | 3.3 |
| Ankles-only | 0.63 | 0.46 | 0.41 | 0.5 |

### RMS applied torque when zero-torque is commanded for each joint and participant

| | P1 | P2 | P3 |
|---|---|---|---|
| RMS torque (Nm) at knees and ankles during hip-only | 0.67 | 0.54 | 0.86 |
| RMS torque (Nm) at hips and ankles during knee only | 0.66 | 0.57 | 0.41 |
| RMS torque (Nm) at at hips and knees during ankle-only | 0.91 | 0.69 | 0.84 |

## 14. Pilot tests

Before the single-joint optimization experiments of this study, we first conducted a pilot experiment trying to optimize whole-leg assistance with fewer parameters. In this optimization we included nine parameters in total to define the hip, knee and ankle torque profiles. The intention was to keep this parameter number small to ensure convergence. We optimized hip flexion rise time, hip flexion peak torque magnitude, hip flexion peak torque timing, hip extension peak torque magnitude, hip extension peak torque timing, knee flexion torque, knee stiffness, knee damping, peak ankle torque magnitude, and peak ankle/knee flexion torque timing (the two peaks were constrained to be at the same time). The other parameters that we included in subsequent optimizations were fixed in this experiment, with values based on hand-tuning.

We optimized this assistance for P1 for 30 generations over 10 days. This was intended to be long enough to ensure training and convergence.

When the assistance was validated, the nine-parameter hip-knee-ankle assistance reduced the metabolic cost of walking by only 20% relative to walking in the exoskeleton with no torque applied. The participant also found the profile uncomfortable. We knew because we had seen ankle-only assistance that reduced the metabolic cost of walking by more than 20%, that better assistance should be attainable. We decided that more parameters would be necessary to allow the optimizer to fully explore all possible strategies. Instead of going straight to the 22 parameter whole-leg assistance optimization, we decided to optimize single-joint strategies first to optimize smaller parameter sets and to ensure that our device was able to apply effective assistance similar to previous devices.

## 15. Detailed optimization methods

For an introduction to CMAES as well as good pseudocode, we recommend the CMA-ES wikipedia page.

*Initial sigma values*

The sigma value in the CMA-ES optimization adjusts the step size and the size of the distribution from the sample that is taken. It is defined for the first generation and updated by the algorithm when calculating the next generation. Larger sigmas mean more exploration, and it is expected to shrink as the optimizer converges on the optimum parameters. Sigma was initialized as 0.15 for optimization of single-joint assistance for P1 to ensure a wide-enough search to find optimal assistance. Sigma was initialized as 0.1 for optimization of whole-leg assistance for P1, since we expected that the initial values for the search (which were based on single-joint optima) were close to an optimal whole-leg assistance strategy. Sigma was initialized as 0.1 for all optimization for P2 and P3, since we expected that the initial values for the search (which were based on P1's optimized assistance) would be close to optimal assistance strategies for P2 and P3. Parameters in the optimization were normalized to the range of 0 to 1, so that this sigma is uniform across all parameters.

*Number of conditions per generation*

The number of conditions per generation, called *lambda*, was a function of the number of parameters being optimized *cmaesN,* according to this formula:

lambda=4+floor(3*log(cmaesN))

This means that hip-only and knee-only had 10 conditions per generation, ankle-only had 8, whole-leg had 13, hip-knee had 12, and hip-ankle and knee-ankle had 11 conditions per generation.

*Elitism and means in each generation*

When creating a generation, the optimizer sampled (*lambda* - 2) conditions from the distribution based on the current means and covariance matrix. We then set the last condition of the generation to be the parameter means for that generation, and the second to last condition to be the "elite" condition from the last generation, meaning the condition that resulted in the lowest metabolic score. This was done to improve convergence by biasing the optimizer to more exploitation than exploration, and also was intended to make sure the optimizer didn't drift away from any well-performing conditions.

*Handling of constraints*

If parameters were sampled outside of our constraints, the optimizer saturated the value and applied the constraint value instead, but it allowed the mean of the generation to drift outside of the constraints.

## 16. Tested parameter values during optimization

### Hip-only

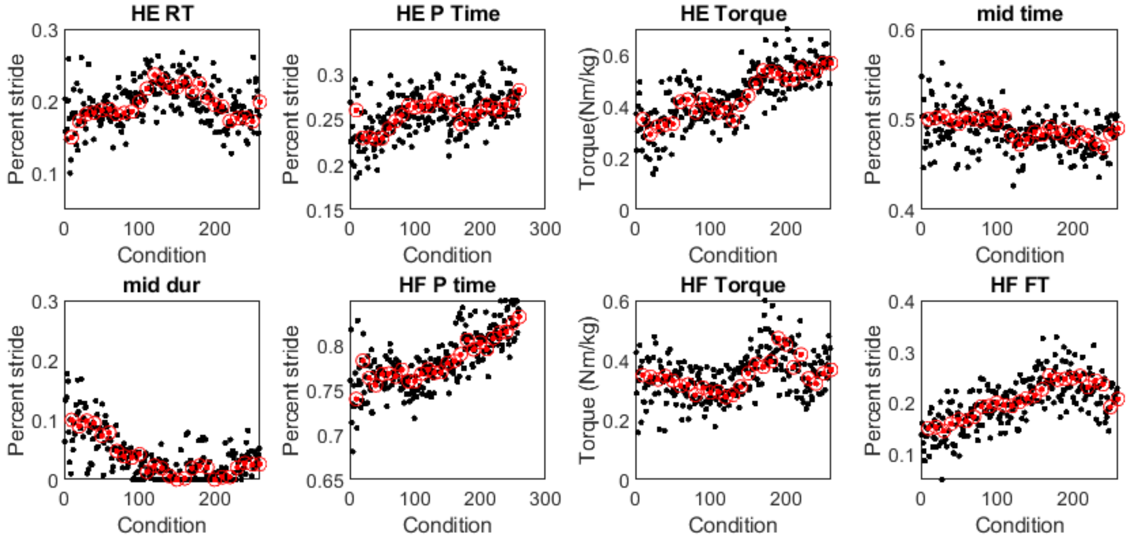


### Knee-only

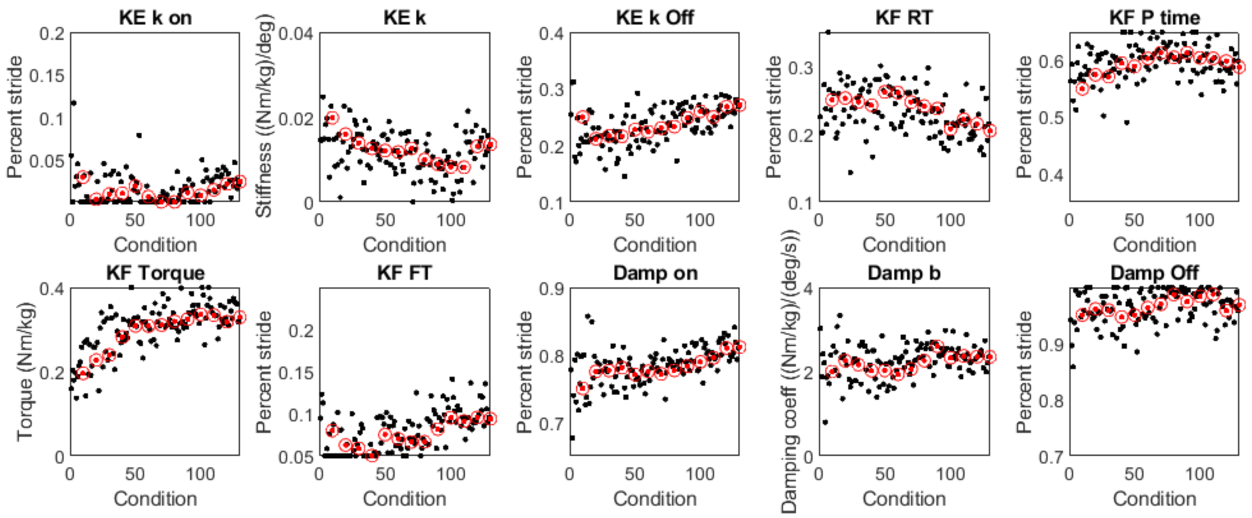


### Ankle-only

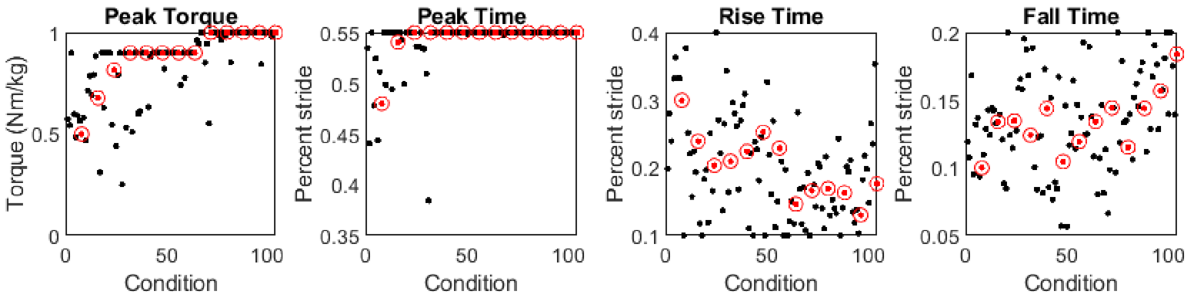


**Parameters tested during single-joint optimization.** Tested parameter values for each condition during optimization of each single-joint assistance for participant 1. The mean value for each generation is shown in red. Y-axis bounds are the minimum and maximum allowed parameter values. As the algorithm converges on an optimum parameter value we expect the distribution to shrink and for the values tested to level off. Wide sample distributions and large changes in tested values could mean the optimizer is still searching, or it could mean this parameter did not have a large effect on our optimized cost function, namely metabolic cost.

## 17. COVID mask effect on metabolics

**Participant 1 metabolic cost measurements with and without a cloth mask, tested on separate days**

| (W/kg) | Quiet Standing | No Exo. | No Torque |
|---|---|---|---|
| No cloth mask | 1.43 +/- 0.09 | 4.09 +/- 0.15 | 4.66 +/- 0.14 |
| Cloth mask | 0.84 +/- 0.07 | 2.71 +/- 0.02 | 3.30 +/- 0.16 |
| Absolute change (no mask vs. mask) | -0.59 | -1.38 | -1.36 |
| % change (no mask vs. mask) | -41% | -34% | -29% |

For all two-joint optimizations except for P3 hip-ankle optimization and validation, a cloth mask was worn underneath the metabolics mask to comply with safety protocols required due to the COVID-19 pandemic. This cloth mask affected the accuracy of the metabolics measurements. The metabolic scores decreased when the participant wore a cloth mask. For the metabolic conditions reported in this study, the cost of standing quietly was subtracted from the measured cost of the walking conditions. This would mean that any constant offset error in metabolic cost due to the mask would be controlled for by subtracting the cost of quiet standing. We expect some error on metabolic cost measurements that is proportional to metabolic cost due to increased breathing rates.

We expect that the cloth mask made it difficult to have a tight seal between the user's face and the metabolics mask. This means air could possibly be seeping between the room and within the mask. This could mean that air from the room could be getting into the sampler that measures $O_2$ and $CO_2$, which could affect the metabolic measurement by decreasing the relative amount of $CO_2$ measured, which would lead to a decrease in the reported metabolic rate. Similarly, a leaky seal between the metabolics mask and the user could mean that air during breathing could leak out, which could cause the turbine to underestimate the volume of breath, also leading to an underestimation of metabolic cost. We believe that these two effects could be roughly proportional to breathing rate, which in turn is roughly proportional to metabolic cost. For this reason, we think the effect of the cloth mask underneath the metabolics mask has a percentile effect on the measured metabolic cost. Because we are comparing in between conditions and calculating percent metabolic cost reductions, we believe the relative reductions to still be accurate, while the absolute predictions of metabolic cost to be noisier. This cloth-mask effect only impacts the measurements of two-joint assistance.

## Participant 2 metabolic cost measurements with and without a cloth mask, tested in same experiment

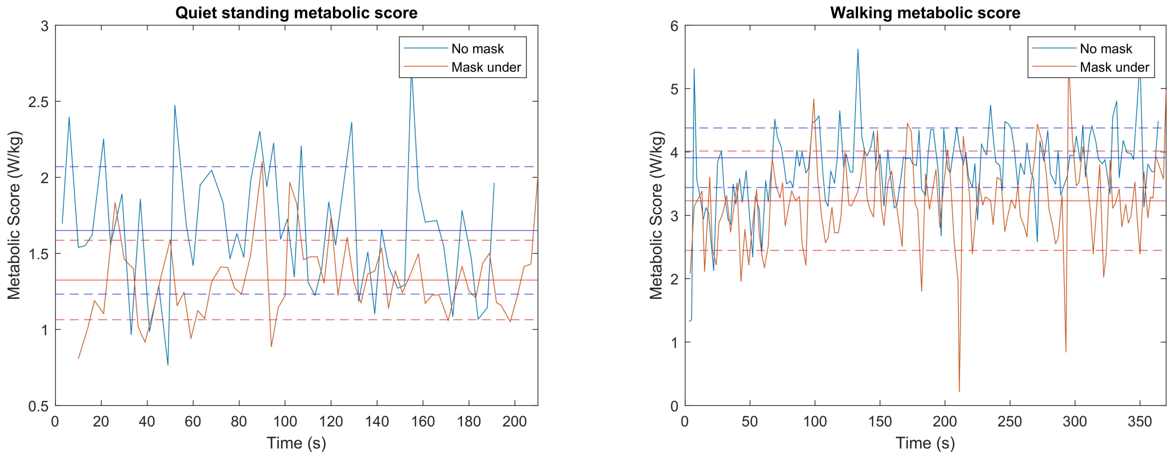


Metabolic cost as a function of time during standing (left) and walking without an exoskeleton (right) without a cloth mask (blue) and while wearing a cloth mask under the metabolics mask (red). Breath by breath measurements are shown, as well as the average value shown as a solid horizontal line with plus and minus one standard deviation shown with dotted lines. This test further demonstrates that the cloth mask has a downward effect on estimated metabolic cost.
